# Seabirds show foraging site and route fidelity but demonstrate flexibility in response to local information

**DOI:** 10.1186/s40462-024-00467-9

**Published:** 2024-06-13

**Authors:** Charlotte E. Regan, Maria I. Bogdanova, Mark Newell, Carrie Gunn, Sarah Wanless, Mike P. Harris, Samuel Langlois Lopez, Ella Benninghaus, Mark Bolton, Francis Daunt, Kate R. Searle

**Affiliations:** 1 UK Centre for Ecology & Hydrology, Bush Estate, EH26 0QB Penicuik, Midlothian, UK; 2 RSPB Centre for Conservation Science, AB15 6GZ Aberdeen, UK

**Keywords:** Atlantic puffin, Black-legged kittiwake, Common guillemot, Foraging route fidelity, Foraging trip, Individual foraging site fidelity, Razorbill

## Abstract

**Background:**

Fidelity to a given foraging location or route may be beneficial when environmental conditions are predictable but costly if conditions deteriorate or become unpredictable. Understanding the magnitude of fidelity displayed by different species and the processes that drive or erode it is therefore vital for understanding how fidelity may shape the demographic consequences of anthropogenic change. In particular, understanding the information that individuals may use to adjust their fidelity will facilitate improved predictions of how fidelity may change as environments change and the extent to which it will buffer individuals against such changes.

**Methods:**

We used movement data collected during the breeding season across eight years for common guillemots, Atlantic puffins, razorbills, and black-legged kittiwakes breeding on the Isle of May, Scotland to understand: (1) whether foraging site/route fidelity occurred within and between years, (2) whether the degree of fidelity between trips was predicted by personal foraging effort, and (3) whether different individuals made more similar trips when they overlapped in time at the colony prior to departure and/or when out at sea suggesting the use of the same local environmental cues or information on the decisions made by con- and heterospecifics.

**Results:**

All species exhibited site and route fidelity both within- and between-years, and fidelity between trips in guillemots and razorbills was related to metrics of foraging effort, suggesting they adjust fidelity to their personal foraging experience. We also found evidence that individuals used local environmental cues of prey location or availability and/or information gained by observing conspecifics when choosing foraging routes, particularly in puffins, where trips of individuals that overlapped temporally at the colony or out at sea were more similar.

**Conclusions:**

The fidelity shown by these seabird species has the potential to put them at greater risk in the face of environmental change by driving individuals to continue using areas being degraded by anthropogenic pressures. However, our results suggest that individuals show some flexibility in their fidelity, which may promote resilience under environmental change. The benefits of this flexibility are likely to depend on numerous factors, including the rapidity and spatial scale of environmental change and the reliability of the information individuals use to choose foraging sites or routes, thus highlighting the need to better understand how organisms combine cues, prior experience, and other sources of information to make movement decisions.

**Supplementary Information:**

The online version contains supplementary material available at 10.1186/s40462-024-00467-9.

## Background

Phenotypic variation within populations is both widespread and critical in shaping population, community, and ecosystem processes [1]. Individual specialisation often manifests as specialised foraging behaviour, with individuals frequently displaying preferences for specific habitat or prey [2, 3], or repeatedly using the same location or route of travel (i.e., exhibiting fidelity) [4–6]. Fidelity to a specific location or route is expected to be advantageous when the environment is stable or predictable [7], though it may also prove beneficial in unpredictable environments where familiarity is advantageous, such as in predator avoidance, maintaining social relationships, or avoiding costly movements [8].

Fidelity, may however, have negative consequences for individuals when it drives them to continue to use areas that are no longer predictable or whose value has decreased [9]. Human activity is leading to such changes in habitats across the globe, both over short and long timescales [10]. In the face of such changes, strong fidelity may lead to individuals continuing to use areas that are declining in value or increasing in risk, and thereby reducing their prospects of survival and/or reproductive success [9]. Such effects on individual fitness may, in turn, have knock-on consequences for the resilience and viability of populations [9, 11, 12]. Therefore, understanding the magnitude of fidelity in different species, as well as its drivers, will aid the development of robust mitigation and conservation strategies.

The degree of fidelity shown by individuals to a specific location may be driven by multiple processes, relating to both individual and population characteristics, as well as environmental variables. At its root, fidelity is expected to be high when environments are temporally and spatially predictable and individuals locate high quality sites and re-visit them while avoiding other lower quality ones [13]. In this situation, individuals would be expected to show high site fidelity until conditions deteriorate at which point, they will switch to an alternative site (the so-called ‘win-stay, lose-switch’ strategy, sensu [7]). However, the degree to which an individual displays fidelity will also depend on other factors, such as their prior experience and memory of spatial information [14–16] and their relative use of personal versus social information [17]. Although work has begun to understand some specific drivers of fidelity, such as the effect of environmental predictability and prior success [18], we still have little understanding of how individuals balance the information they gain from their own foraging activities against other sources of information, such as large scale environmental cues and the information they may gain by observing and interacting with others (but see [17–19]). Closing this gap will facilitate better predictions of how animals will respond to human induced environmental changes.

Colonial breeding seabirds are a useful study system for asking questions about foraging fidelity and its drivers given that (i) their ties to a breeding site impose strong time and space constraints on foraging, potentially making fidelity very advantageous under predictable environmental conditions; (ii) rich GPS datasets combined with data on foraging behaviour provide an opportunity to study the dependence of fidelity on foraging effort and (iii) the close proximity of individuals at and around the colony may provide opportunities for individuals to observe the foraging behaviour and success of con- and heterospecifics and subsequently use this information to determine their foraging behaviour [20]. Fidelity has been quantified in some seabird species (e.g. northern gannet, *Morus bassanus* [21]; black-legged kittiwake, *Rissa tridactyla* [22]. However, studies to date have been limited by notably narrow taxonomic breadth, commonly short timescales [23, 24], and a focus on foraging areas rather than entire foraging trips. Further, little is known about the processes that drive or erode fidelity.

In this study, we use long-term GPS tagging data for four seabird species breeding on the Isle of May National Nature Reserve, in South-East Scotland, to estimate the degree of fidelity over entire foraging routes (i.e., including travel to and from foraging grounds) and to understand if individuals flexibly adjust their routes in response to information gained when foraging (i.e. personal information) as well as local environmental cues/the presence or behaviour of other individuals at/near the colony or foraging out at sea. We first quantified the degree of foraging site/route fidelity within- and between-years and whether fidelity eroded over time. Second, we assessed whether the degree of foraging site/route fidelity was explained by feeding behaviour on the previous trip. Finally, we investigated whether individuals exhibited more similar trips when they were at the colony or at sea at the same time, suggesting the use of a common environmental cue or information on the foraging routes and/or success of other individuals when choosing their foraging routes.

## Methods

### Study system and GPS tagging

The study was carried out on the Isle of May National Nature Reserve (56°11’N, 2°33’W) using GPS data for four seabird species: common guillemots (*Uria aalge*), Atlantic puffins (*Fratercula arctica*), razorbills (*Alca torda*), and black-legged kittiwakes. These seabirds feed on small, shoaling, forage fish during chick-rearing (see Figure S1 for foraging ranges of these species breeding on the Isle of May), and though marine environments are strongly heterogeneous these prey resources are predictable in temperate and polar regions, relative to the tropics, due to particular features such as frontal zones, upwellings, and shelf edges [25]. Seabirds appear to have a good knowledge of the location and concentrations of patches in these regions and generally use a commuting type of trip to reach foraging zones [25]. In these foraging environments oceanographic features may concentrate prey species and provide seabirds with a spatially and temporally predictable food supply, as is the case with frontal systems in the North Sea and tidal cycles in the Celtic Sea [26, 27]. A study of the Wee Bankie region in our study area demonstrated that black-legged kittiwakes use foraging habitat characteristics that were predictable, albeit sparse and patchy [28].

Alternatively, such concentrations of prey may result from interactions with other predators leading to local enhancement via multi-species predator assemblages [26, 29]. Here, groups of guillemots or razorbills may drive a shoal of fish towards the surface exploiting prey from below, where kittiwakes may then detect the foraging assemblage and exploit the prey from above [30]. In our study area, previous work has shown that foraging associations of kittiwakes and guillemots tend to form in particular areas [28], suggesting a degree of predictability in prey resources. High densities of foraging kittiwakes typically occurred in areas where such multi-species foraging assemblages were both large and frequent, demonstrating the role of prey facilitation by diving taxa in this surface feeding species [28].

The four species we focus on in this study breed in large colonies, with guillemots, razorbill, and kittiwakes nesting on cliff ledges, whilst puffins rear their chicks in burrows. All rely particularly heavily on sandeels (*Ammodytes* spp.) during the breeding season, though the diet composition in the Isle of May populations has changed over time, particularly in guillemots where there has been a marked shift towards sprat (*Sprattus sprattus*) in the diet of chicks [31]. Puffins, guillemots, and razorbills are all pursuit divers that carry fish in the beak back to the chick, whilst kittiwakes are surface feeders that regurgitate semi-digested fish to their chicks. Although these species exploit the same prey resource, the flight costs they experience varies due to differences in wing-loading, with costs being lower in kittiwakes than in the three auk species, particularly common guillemots [32, 33]. Such differences in flight costs may alter the degree to which fidelity is beneficial and thus the patterns of fidelity observed in the four species. The species in this study are known to form multi-species foraging aggregations out at sea [30], with guillemots and razorbills being key initiators of such feeding frenzies. There is also experimental evidence that guillemots and puffins are attracted to aggregations of con- and hetero-specifics [34]. Prior work has also suggested the potential for seabird colonies to act as information transfer hotspots [20]. Thus, seabirds may access social information about the location and profitability of foraging areas both at the colony and at sea and adjust their foraging routes in response to this information, though evidence for this is limited.

Across five to eight years between 2010 and 2021, adults of the four species were tagged with GPS devices mostly when feeding young (see Table S1 for details on capture methods, tag types, tagging years, deployment durations, GPS sampling schedules, and sample sizes for each species). Across one to ten capture areas in each year (see Figure S2 and Table S1 for details), individuals were captured at the nest site with a noose at the end of an extendable pole, or in the case of puffins, in the burrow or by purse or mist nets at the burrow entrance. GPS devices were attached to the back or tail feathers of kittiwakes using waterproof Tesa tape or superglue, whereas in the other species the device was attached to back feathers using waterproof Tesa tape (Table S1). From 2010 to 2014, archival tags were used so individuals had to be recaptured and devices removed so that the data could be downloaded. From 2018 to 2021, data were remotely downloaded to base stations when individuals were at the colony. Tags varied in their sampling schedules between species and years, with fixes mainly collected between every one and every ten minutes (see Table S1 for details). The mean number of days that individuals were tracked varied between years due to differences in the tags used and in sampling schedules (puffin: 1.76 days– 6.14 days, guillemot: 1.75 days– 6.78 days, razorbill: 1.22 days– 5.95 days, kittiwake: 1.25 days − 17.24 days).

Puffins are known to be sensitive to logger attachment and previous work on the Isle of May has shown that logger attachment affects chick provisioning behaviour and thus chick weights and breeding success [35, 36]. In response to these findings, we have used the smallest available loggers, ensured only one bird per breeding pair was tagged, and have used supplementary feeding to bolster chicks against tagging impacts. Nevertheless, it is possible that the foraging behaviour of the puffins featured in this study may not be representative of untagged individuals. We also found some evidence for device effects on kittiwakes tagged with UvA-BiTS loggers (22 of 22 individuals were tagged with these loggers in 2020 and 13 of 50 in 2021), with fewer parent changeovers at the nest, indicative of longer foraging trips, and lower chick attendance for pairs where one individual was deployed with a UvA-BiTS logger compared to controls, though these differences did not translate into differences in chick condition and breeding success [36]. Importantly, comparisons of foraging trip metrics and space use of individuals tagged with UvA-BiTS loggers with those tagged with Pathtrack loggers suggests that the use of UvA-BiTS loggers did not alter foraging behaviour of individuals breeding on the Isle of May [36].

Raw data from GPS devices were processed using R (version 4.1.0 [37]). First, fixes recorded prior to a bird’s release and after its recapture were removed, and any fixes in improbable locations (i.e., far outside of the foraging area of these species) were excluded. Second, where there were fixes with duplicate timestamps (< 1.1% of fixes in all cases), a single fix was randomly selected. We opted for this approach as it was unclear which of the fixes was ‘real’. Third, for IgotU tags (used from 2010 to 2014), where there were instances of consecutive fixes being identical in location, altitude, bearing, and speed, but having different timestamps, we excluded the second fix in each duplicate pair as this pattern is extremely unlikely to be real (from < 1% of fixes up to 7.9% of fixes across species and years). Finally, we excluded fixes occurring immediately after movements where speeds exceeded 30 m s^− 1^ to remove fixes caused by GPS error (< 1% of fixes in all cases). We chose a 30 m s^− 1^ cut-off based on the distributions of flight speeds over all tracking years for each species (see Figure S3). This cut-off ensures the inclusion of biologically realistic flight speeds (mean flight speeds - razorbill = 16.0 m s^− 1^, guillemot = 19.1 m s^− 1^, puffin = 17.6 m s^− 1^, kittiwake = 13.1 m s^− 1^; see [38] for details), allowing for higher speeds associated with tail winds whilst excluding biologically implausible flight speeds.

### Trip assignment

We used the ‘tripSplit’ function in the ‘track2KBA’ package (version 1.0.2; [39]) to split tracking data into foraging trips. We classed individual movements from the colony as trips when the bird was >500 m from their capture area for at least 30 min (as in [40]) to exclude movements made for bathing and preening. When fixes were separated by gaps of more than one hour, we split tracks into segments, and carried out trip assignment on each of these segments in turn. Only complete trips (i.e., trips starting and ending at or within 500 m of the colony) were included in subsequent analyses.

### Fidelity metrics

Because fidelity is a potentially complex process, we selected three metrics to describe different aspects of foraging fidelity: (1) the mean nearest neighbour distance between trips (NND; see below for details), (2) the distance between trip distal locations (i.e., the point furthest from the breeding colony), and (3) the difference in bearing between trip distal points. We use ‘foraging route fidelity’ when discussing NND as this uses information over the entire trip, and ‘foraging site fidelity’ when discussing bearing and distal locations, as these metrics focus more on the furthest location reached on a trip, which is often assumed to correspond to the primary feeding location in seabirds (e.g [21, 41, 42]). However, it is important to note that from visual inspection of GPS tracks, it was apparent that individuals sometimes forage at multiple locations during a trip. This would limit our ability to look at fidelity to foraging sites using metrics focused on distal points (i.e., distance between distal points and difference in bearing), but would be captured by the nearest neighbour distance as it considers the whole foraging trip. We sub-sampled trips to 10-minute intervals between fixes prior to calculating fidelity metrics to prevent potential bias in metrics due to different sampling schedules between years and tag types (see Table S1). We selected a 10-minute interval as this was the coarsest resolution at which fixes had been recorded in any of the species/years. We carried out the resampling using the ‘track_resample’ function from the ‘amt’ package (version 0.1.7; [43]), allowing a 60-second tolerance around the 10-minute interval.

**Fig. 1:**
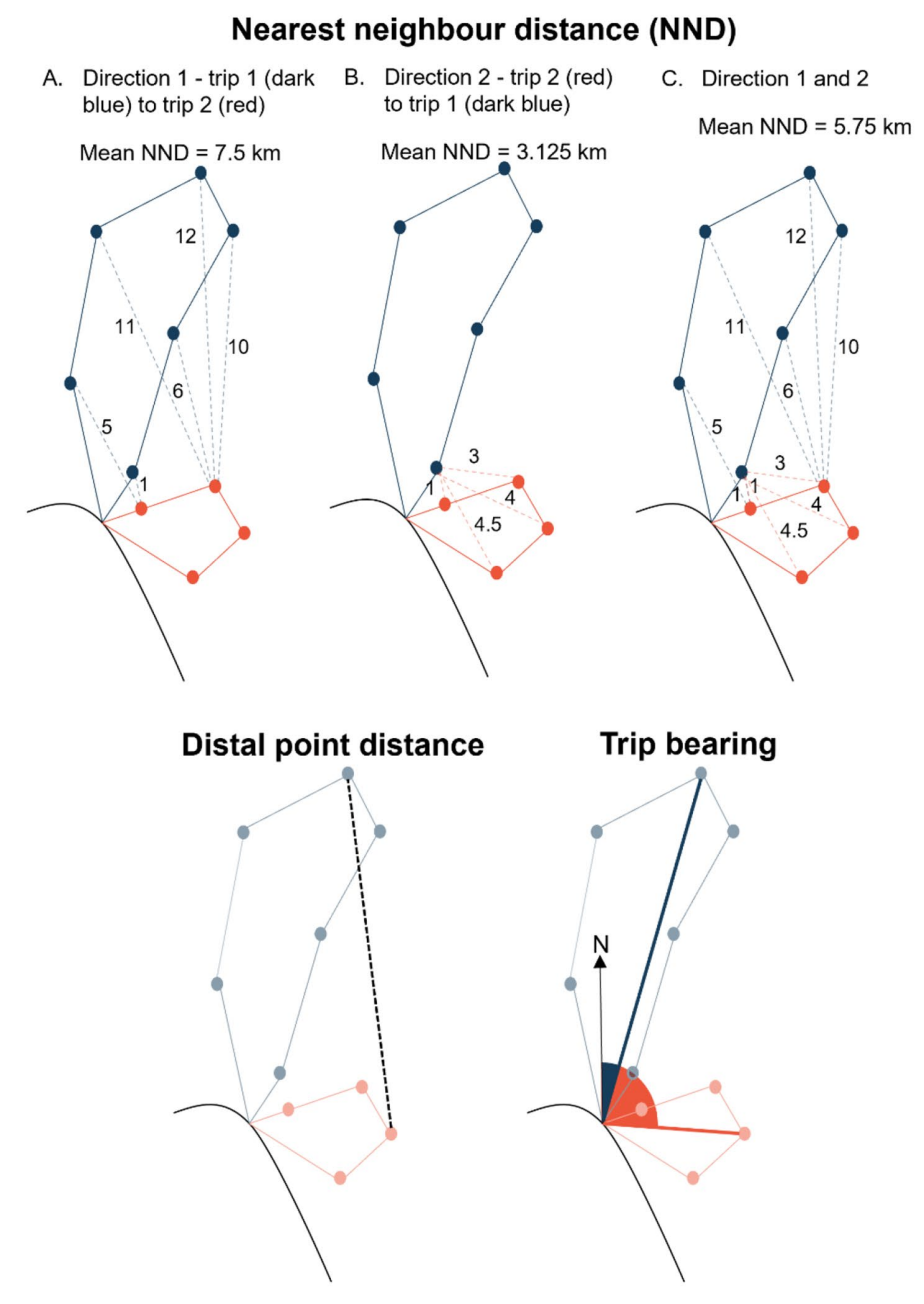
We used 3 metrics to compare foraging trips: mean nearest neighbour distance, the distance between trip distal points and the difference in trip bearing. When comparing trips using the mean nearest neighbour distance (NND), we used fix-level NNDs obtained by comparing trips in both directions (i.e., from trip 1 (blue) to trip 2 (red) and from trip 2 to trip 1). We did this to prevent bias in the NND induced by which of the trips is treated as the focal trip, and because the similarity in trip length as well as position is relevant to our research questions. The bias induced by the order in which trips are compared is shown in panels A and B. When selecting the longer trip (panel A) NNDs are biased upwards, whilst when selecting the shorter trip (panel B) NNDs are biased downwards

We calculated trip bearings, in radians, between the capture area (i.e. breeding site) and a trip’s distal location, using the ‘st_geod_aziumuth’ function in the ‘lwgeom’ package (version 0.2-8; [44]). We then calculated the absolute difference in trip bearing as our metric of the difference in bearing between two trips. To compare distal locations, we calculated the straight-line distance between distal fixes from a pair of trips. Finally, we calculated our trip-level NND by taking the mean nearest neighbour distance for fixes on the focal trip and a comparison trip [45], when comparing trips in both directions (i.e., both trip one to two [Fig. 1A] and from trip two to one [Fig. 1B]). Thus, for each fix on each trip, we calculated the shortest distance to a fix on the other trip, before summing the distances from both comparisons so that our NND metric was not determined by the length of the focal trip and instead captured variation between trips due to both difference in route and length (see Fig. 1C).

**Fig. 2:**
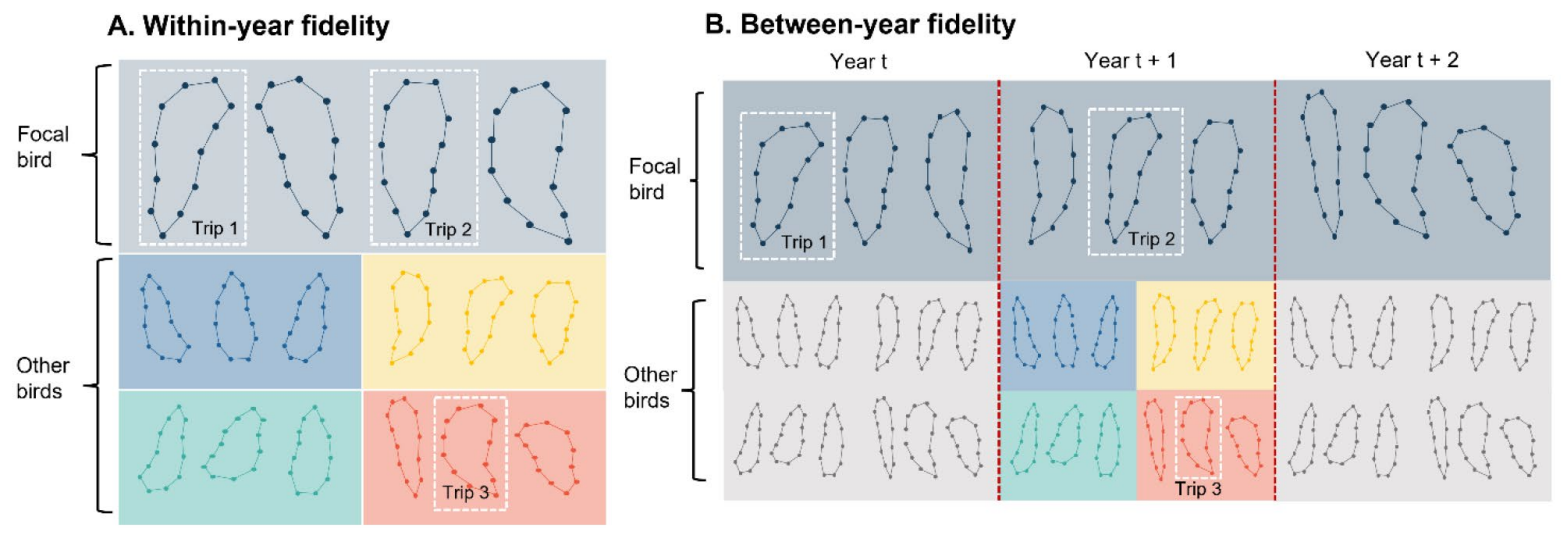
**(A)** To quantify within-year fidelity, we calculated fidelity metrics by comparing a focal trip made by an individual in a given year to another trip made by that same individual in the same year (within-individual comparison), as well as comparing the focal trip to a trip made by a different individual of the same species in the same year (between-individual comparison). **(B)** To quantify between-year fidelity, we calculated fidelity metrics by comparing a focal trip made by an individual in a given year to another trip made by that same individual in a subsequent year (within-individual comparison), as well as comparing the focal trip to a trip made by a different individual of the same species in the same year that the within-individual comparison trip was made (between-individual comparison)

To assess whether there was within-year individual foraging site or route fidelity, we compared the similarity between trips from the same individual to the similarity between trips from different individuals of the same species tagged in the same year (i.e., comparing an empirical versus a null distribution). We did this by both comparing all trips made by the same individual in a given year alongside comparing trips made by the focal individual to a sample of trips made by other individuals of the same species tagged in the same year (Fig. 2A). We took the same number of between-individual samples as there were within-individual samples, such that our analysis would give more weight to individuals with more trip comparisons. To maximise the pool of potential between-individual comparisons, we opted not to match within- and between-individual comparison trips by date. We calculated fidelity metrics for individuals with at least two complete trips in a given year but included complete trips from all other individuals as potential trip comparisons.

When examining between-year site fidelity, we used a similar approach, but compared all trips from each individual in two different years, both consecutive and non-consecutive where available (see Table S2 for data availability summary), as well as comparing a sample of trips from the focal individual and another individual that was tagged in the same year as the second trip from the focal bird (see Fig. 2B). Again, this between-individual sample was the same size as the within-individual sample.

### Measures of foraging behaviour

To investigate whether the fidelity between consecutive trips was explained by foraging effort on the first trip, we used data from time depth recorders (TDR) integrated with the GPS tags deployed on all guillemots and razorbills in 2020 and 2021 (no equivalent foraging data were available for puffins or kittiwakes). Depth was recorded every four seconds once submerged at a depth greater than one metre. We used TDR data to identify foraging bouts within trips by assigning TDR records to the same foraging bout if they were separated by less than five minutes [46–48]. We classified GPS fixes as ‘foraging’ if they fell within assigned foraging bouts. For this purpose, we used GPS data at the original resolution rather than the sub-sampled dataset. For each trip, we quantified foraging behaviour in four different ways: (1) the time between the start of the trip and the start of the first foraging bout as a measure of how long it took individuals to find prey (i.e., a metric of foraging efficiency; [49]), (2) the mean duration of foraging bouts as a measure of the efficiency within bouts, assuming that individuals will remain in a patch for longer when the quality of the patch, and thus their foraging efficiency, is high (following Charnov’s Marginal Value Theorem [48]), (3) the total time spent foraging on a trip under the assumption that longer total time spent foraging across the entire trip corresponds to individuals taking longer to obtain sufficient food for themselves and/or their chick across their trip, and (4) the proportion of time on a trip spent foraging as an alternative measure of foraging efficiency across the trip, with more efficient trips expected to have a lower proportion of time spent foraging. However, the degree to which this indicates high efficiency may vary between species given differences in the costs of flight versus foraging [32, 33]. We must also note that it currently remains unclear how seabird foraging efficiency is most accurately defined and quantified and thus our metrics may still fail to accurately capture foraging trip efficiency. Indeed, there is some evidence to suggest that foraging seabirds will wait at sea for conditions to be most suitable for foraging [51] or will rest for prolonged periods at sea following foraging bouts [52], indicating that foraging trips may not be entirely focused on foraging and returning to the colony as quickly as possible.

### Characterising trip similarity between individuals over short timescales

To understand the degree to which individuals showed similarity in their foraging routes, indicating either the use of common cues or information gained from observing one another, we compared (i) trips made by con- and hetero-specifics on the same day versus on different days, and (ii) trips made by conspecifics that overlapped in time when at the colony versus at sea (see Table 1 for more details). To compare trips made by individuals on the same day versus on different days, we used a similar sampling protocol to above. For each species on each day in each year, we randomly sampled one trip per individual. We compared these trips to each other (within-species, within-day comparison) and to a random sample made by another species on the same day (between-species, within-day comparison). We included trips that spanned more than one day to avoid bias towards shorter trips. We obtained between-day comparisons by pairing our focal trips to trips made by individuals of the same species on a different day (within-species, between-day) and to a random sample of trips made by another species on the same alternative day as the within-species between-day comparison (this formed the between-species, between-day comparison). Thus, we ended up with the same number of within-species and between-species comparisons for each day.

We next compared trips made by individuals that did or did not overlap in time at a given capture area (i.e., breeding site) prior to trip departure under the assumption that overlap would increase the likelihood of individuals being able to assess one another’s return or departure bearing and/or foraging success. We opted not to consider the magnitude of the overlap at the colony as we had no a priori expectation that more time overlapped would translate into the transfer of additional information between specific pairs of individuals. We do acknowledge that the information gained by a focal individual may be correlated with the time spent at the nest due to the potential for observing a larger number of other individuals, but we are currently unable to look at this given that not all individuals are tracked in a given breeding season. To compare trips made by individuals that did or did not overlap in time at the colony (see Table 1 for more details), we took the GPS data for each species separately, and for each capture area on each day. We then randomly sampled one complete trip per individual before comparing trips made by two different individuals using the same fidelity metrics as above (NND, distance between distal points, and difference in bearing). We used an individual’s fixes at the colony prior to leaving on the focal trip to determine whether individuals had overlapped at the colony in the period between their previous trip and the focal trip.

**Table 1:** Questions, metrics, and hypotheses underpinning analyses centred on understanding how foraging trip similarity is predicted by temporal and spatial overlap

Question	Metric	Hypothesis
Do con- and heterospecifics make more similar trips when foraging on the same day?	Comparison of trips made by the same and different species on the same versus different days	Individuals make more similar trips to others foraging on the same day (with conspecifics expected to be more similar than heterospecifics), if they use the same local cues of prey location or availability or foraging behaviour of other individuals to determine their own trips.
Do individuals overlapping at the colony prior to a trip make more similar trips?	Overlap at the colony (yes/no)	Individuals that overlap at the same capture area prior to making a trip will be more similar in their trip if individuals use the same local cues/use information on the direction of departure/return and foraging success of others breeding nearby
Do individuals foraging out at sea at the same time show more similar foraging trips?	The degree of temporal overlap between trips	Individuals overlapping to a greater degree out at sea will be more similar in their trips if individuals use the same local cues of prey location or availability or information on where others are foraging.

We took a very similar approach to determine if the degree of overlap at sea was correlated with trip similarity (see Table 1 for more details), although in this case we allowed comparisons between capture areas as well as within them. Then for each pair of trips made by individuals of the same species on the same day, we calculated the three fidelity metrics (NND, distance between distal points, and difference in bearing) as well as the proportion of total trip time that was shared between the two trips (i.e., time shared/the sum of the two trip durations). When examining the effect of overlap at the colony or at sea, we only considered conspecifics as our comparison of trips made by con- and heterospecifics (see methods above) indicated that trip similarity was significantly higher within species than between species.

### Statistical analysis

#### Do individuals show foraging site and route fidelity and does this vary between individuals and species?

For both within- and between-year analyses, we examined the difference in bearing, distance between distal points, and nearest neighbour distance in turn. In each analysis our response variable was the pairwise trip fidelity metric, and for both the within-year and between-year analyses, we included the difference in trip start times as a fixed effect to control for differences in trip similarity due to the relative timing of trips and to establish whether there was evidence for a decay in fidelity over time. In the within-year analysis, we included the difference (in minutes) between the start of the two trips as our measure of the difference in trip timing.

In the between-year analysis, we included both the number of years separating trips, as well as the difference in the timing of trips relative to the median laying date across all monitoring plots (see Burthe et al. 2012 for details) in their respective years as fixed effects. Due to a lack of laying date information for kittiwakes in 2020 due to the COVID-19 pandemic, we excluded this year for kittiwakes from analyses. We did not carry out a between-year analysis for puffins as only two individuals had been tracked in more than one year (Guillemots = 24 individuals, Kittiwakes = 7 individuals, Razorbills = 5 individuals; Table S2). We included information on the relative timing of trips to control for potential differences in trip characteristics due to individuals being at different stages of the chick-rearing period. Given we used island-level laying date information, this assumes no within-colony variation in laying date. In analyses where nearest neighbour distance or the distance between distal locations was the response, we included the straight-line distance between capture areas (in metres) as a fixed effect to control for differences in the apparent similarity of trips due to the nest locations of comparison individuals.

For each species, we used two models to quantify (1) whether individuals showed foraging site or route fidelity and (2) whether the degree of fidelity varied depending on trip timing. The first model included the comparison type (i.e., within-individual or between-individual) to test whether trip similarity was significantly higher within individuals versus between-individuals, thus signifying fidelity. The second model also included an interaction between the comparison type and the time difference between trips to determine whether the difference between within-individual and between-individual comparisons (i.e., the degree of fidelity) varied according to how close together trips were in time, and hence if fidelity varied over time. When considering between-year fidelity we used the difference, in years, between trips as the measure of timing in this interaction. In the within-year analysis, we included individual identity nested within tracking year as random effects to estimate the degree to which fidelity differed between individuals and years, and to avoid pseudo-replication. Similarly, in the between-year analysis, we included individual identity as a random effect to account for potential differences in fidelity among individuals.

#### Is within-year fidelity explained by foraging behaviour?

We examined the relationship between our measures of guillemot and razorbill foraging behaviour (time to first foraging bout, mean foraging bout length, total time spent foraging, and proportion of time spent foraging) and the fidelity between consecutive trips using each fidelity metric in turn (i.e., NND, distance between distal points, and difference in trip bearing). We did this separately for razorbills and guillemots but used the same model structure in each case. In each model, we included the tagging year (two-level factor), the difference between trip start times (in minutes), and each of the foraging metrics as fixed effects as there was no evidence for collinearity between these terms, with all variance inflation factors less than two. We also included individual identity as a random effect to account for potential differences in fidelity between individuals.

#### Characterising trip similarity between individuals over short timescales

To estimate whether individuals tend to have more similar foraging routes to con- and hetero-specifics on a given day, suggesting either the use of a common cue or of information gathered from observing others, we compared trips made by con- and hetero-specifics on the same day versus on different days. For each species and fidelity metric in turn, we performed separate within-species and between-species analyses. The within-species model included the comparison type (i.e., within-day versus between-day) and the distance between capture areas to account for potential differences in trip similarity due to differences in trip start location. The between-species analysis consisted of two models. The first included the same fixed effects as the within-species analysis but also included the comparison species. The second model included the same terms but also featured an interaction between comparison type and comparison species to test whether the difference between within-day and between-day comparisons varied depending on the species being compared. Both within-species and between-species models included year as a random effect.

We next asked whether individuals that overlapped at the colony prior to making their trips were more similar in their trips than those that did not. To do this, we only compared trips of individuals that were captured, and thus nesting, in the same capture area and thus had the potential to observe each other when at the nesting area and/or on approach or departure. For each species, we used a model with one of the three fidelity metrics as the response and colony overlap (two-level factor: overlapped or did not overlap) as the single fixed effect. In each model, we included the comparison day nested within tracking year as random effects to account for potential differences in trip similarity between days and years.

Finally, we asked if trips were more similar when they overlapped more in time. For each species, we used a mixed effects model with the same fidelity metrics as above as the response and the proportion of total trip time that was shared between the two trips and the distance between capture areas (except for puffins as all individuals were from the same capture area) as fixed effects. We excluded trips with zero temporal overlap as such trips could be separated in time by only minutes or several hours making them difficult to compare. As in the analysis examining the effect of overlap at the colony, we including day nested within tracking year as random effects.

All covariates were mean centred and scaled to enable direct comparison of effect sizes. NND, distance between distal points, and the difference in trip bearing were all square root transformed prior to analysis to meet the assumption of residual normality. All analyses were conducted using the package ‘lme4’, assuming Gaussian errors.

## Results

We used data from 531 individual seabirds (169 guillemots, 195 kittiwakes, 92 razorbills, and 75 puffins) tracked for between 5 and 8 years between 2010 and 2021. Tracking deployments lasted an average of 4.6 days (min = 0.04 days, max = 36.7 days) and individuals of each species made on average one or two trips per day (guillemot median = 1, kittiwake, puffin, and razorbill median = 2), though sometimes individuals were recorded making between five and seven trips in a 24-hour period (guillemot and puffin max = 6, kittiwake = 5, razorbill = 7). In guillemots and puffins, trips lasted just over 8 h on average (guillemot = 8.81 h, puffin = 8.71 h). Trips made by kittiwakes and razorbills were shorter on average at 6.46 h and 5.37 h respectively.

### Do individuals show foraging site or route fidelity and does this vary between individuals and species?

#### Within-year fidelity

We found strong evidence for within-year fidelity in individuals of all four species during the chick rearing period. Within-individual distances between distal points on trips and within-individual NNDs were substantially smaller than those from between-individual comparisons (Table 2, see Table S3 for full model outputs). This suggests that foraging locations (as indicated by distal points) and complete trips were more similar within individuals than between different individuals of the same species tracked in the same year. We also found that the within-individual difference in trip bearing was smaller than the between-individual difference in bearing for all species, though for puffins the effect was markedly smaller than in the other three species indicating that trips made by the same individual were no more similar in bearing than trips made by different individuals (Table 2 and S2).

There was strong evidence that individuals varied in the degree of fidelity they displayed within a year when examining NNDs (guillemot - χ²(1) = 119.75, p = < 0.0001, kittiwake - χ²(1) = 429.89, p = < 0.0001, razorbill - χ² (1) = 113.69, p = < 0.0001, puffin - χ² (1) = 21.52, p = < 0.0001), distal points (guillemot - χ² (1) = 168.59, p = < 0.0001, kittiwake - χ² (1) = 664.94, p = < 0.0001, razorbill - χ² (1) = 117.06, p = < 0.0001, puffin - χ² (1) = 30.91, p = < 0.0001), and bearings (guillemot - χ² (1) = 300.46, p = < 0.0001, kittiwake - χ² (1) = 1741.51, p = < 0.0001, razorbill - χ² (1) = 170.32, p = < 0.0001, puffin - χ² (1) = 151.70, p = < 0.0001).

**Table 2:** Effect size estimates and associated 95% confidence intervals from the within-year fidelity analyses for each of the four species

	Within individual Est (95% CI)	Between individual Est (95% CI)	*P* value
**Nearest neighbour distance**			
Guillemot	9.10 km (7.54–10.80)	14.78 km (12.77–16.93)	< 0.001
Kittiwake	12.71 km (11.48–14.01)	15.94 km (14.55–17.39)	< 0.001
Razorbill	9.95 km (7.28–13.02)	13.83 km (10.65–17.42)	< 0.001
Puffin	11.53 km (9.73–13.49)	16.58 km (14.39–18.92)	< 0.001
**Distal point distance**			
Guillemot	16.71 km (13.83–19.86)	27.15 km (23.44–31.13)	< 0.001
Kittiwake	27.29 km (24.94–29.75)	34.04 km (31.40–36.78)	< 0.001
Razorbill	19.56 km (14.96–24.77)	26.74 km (21.30–32.79)	0.063
Puffin	18.58 km (17.36–19.85)	26.02 km (24.57–27.51)	< 0.001
**Bearing difference**			
Guillemot	53.75$^\circ$ (45.27–62.95)	77.90$^\circ$ (67.67–88.84)	< 0.001
Kittiwake	37.59$^\circ$ (26.90–50.08)	44.39$^\circ$ (32.69–57.87)	< 0.001
Razorbill	61.71$^\circ$ (55.02–68.77)	75.37$^\circ$ (67.99–83.12)	< 0.001
Puffin	39.58$^\circ$ (35.15–44.27)	41.17$^\circ$ (36.65–45.96)	0.326

**Fig. 3:**
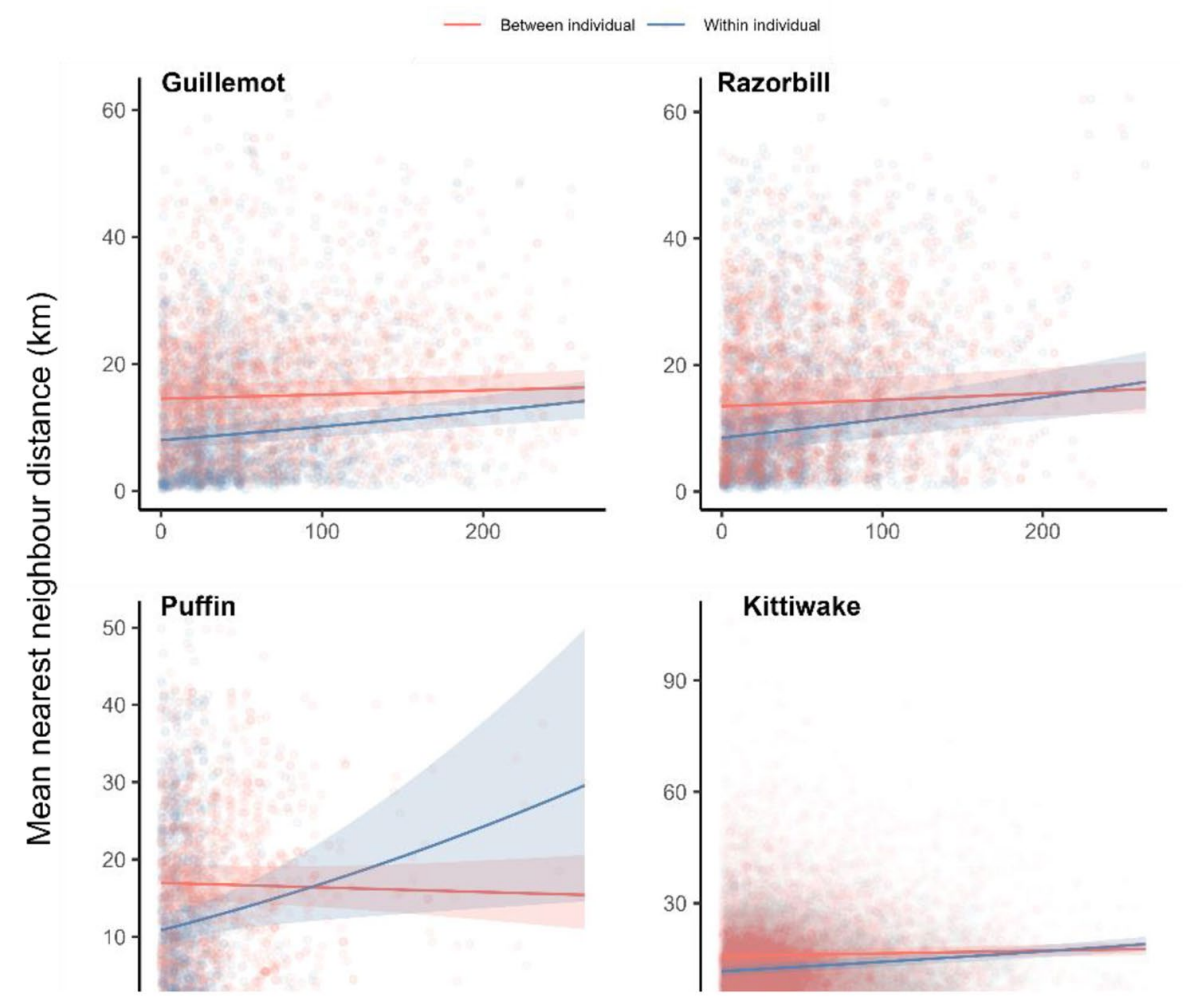
Relationships between the mean nearest neighbour distance between two trips and the time between trip starts. Shown are the raw data (points) and model predictions with associated 95% confidence intervals

In the case of the NND and distance between distal points, the difference between within-individual and between-individual trip comparisons decreased as trips were more separated in time in all species, suggesting that fidelity decreases over time (Fig. 3, Table S4). In kittiwakes and razorbills, the difference between within-individual and between-individual comparisons of trip bearings also decreased over time. However, in guillemots and puffins, there were no contrasting temporal trends in within-individual and between-individual comparisons (Table S4), suggesting within-individual comparisons tended to be more similar in their bearing than between-individual comparisons, regardless of how far apart the trips were in time.

#### Between-year fidelity

**Fig. 4:**
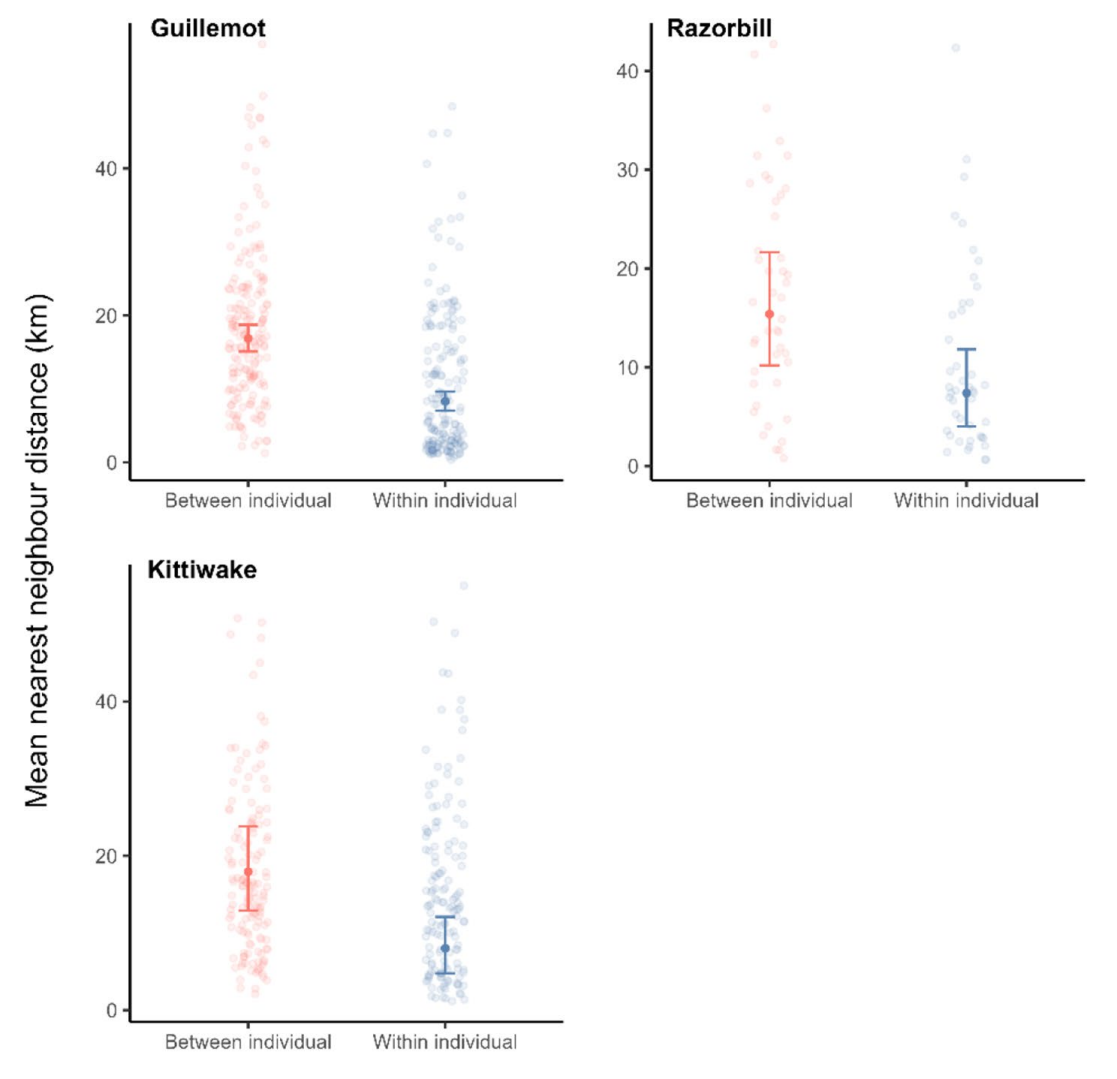
The mean nearest neighbour distance between trips made in different years by the same individual (within-individual) and different individuals (between individual). Shown are the raw data and model predictions with associated 95% confidence intervals

We also found evidence for fidelity across years, though our results somewhat depended on the fidelity metric used. When using NND, within-individual comparisons were more similar than between-individual comparisons in guillemots, razorbills, and kittiwakes, suggesting that fidelity persists within individuals across years (Fig. 4, Table S5). When using both the distance between distal points and the difference in trip bearing, within-individual comparisons tended to be more similar than between-individual comparisons across years, suggesting fidelity persists over time. However, the difference was relatively small, particularly for kittiwakes and razorbills (Table 3, see Table S5 for full model outputs).

In the case of between-year fidelity, when looking at NNDs we only found evidence for significant differences between individuals in the degree of fidelity across years for razorbills (χ² (1) = 7.17, *p* = 0.007) and kittiwakes (χ² (1) = 4.36, *p* = 0.037). In the case of distal point distances, there was evidence for significant differences between individuals inthe degree of fidelity across years in both guillemots and razorbills (guillemot - χ² (1) = 9.00, *p* = 0.003, kittiwake - χ² (1) = 0.008, *p* = 0.927, razorbill - χ² (1) = 17.91, p = < 0.0001). For bearings, we only found evidence that individuals differed in the fidelity they displayed across years in guillemots and razorbills (guillemot - χ² (1) = 18.74, p = < 0.0001, kittiwake - χ² (1) = 3.52, *p* = 0.06, razorbill - χ² (1) = 7.20, *p* = 0.007).

**Table 3:** Effect size estimates and associated 95% confidence intervals from the between-year fidelity analyses for each of the three species considered in this analysis

	Within individual Est (95% CI)	Between individual Est (95% CI)	*P* value
**Nearest neighbour distance**			
Guillemot	8.31 km (7.08–9.64)	16.87 km (15.10–18.74)	< 0.001
Kittiwake	10.36 km (3.55–20.75)	20.64 km (10.40–34.36)	0.005
Razorbill	7.41 km (4.02–11.82)	15.39 km (10.19–21.65)	< 0.001
**Distal point distance**			
Guillemot	21.20 km (17.98–24.67)	31.23 km (27.19–35.55)	< 0.001
Kittiwake	23.28 km (15.19–33.09)	32.51 km (22.98–43.68)	0.159
Razorbill	22.52 km (13.33–34.11)	26.39 km (16.23–39.02)	0.261
**Bearing difference**			
Guillemot	51.91$^\circ$ (39.55–65.95)	79.52$^\circ$ (63.60–97.21)	< 0.001
Kittiwake	24.76$^\circ$ (8.70–49.02)	34.97$^\circ$ (15.48–62.28)	0.342
Razorbill	69.05$^\circ$ (41.68–103.31)	81.11$^\circ$ (50.63–118.75)	0.396

### Is within-year fidelity explained by foraging behaviour?

For both guillemots and razorbills, the degree of fidelity between consecutive trips was significantly associated with foraging behaviour on the initial trip. When using the NND as the fidelity metric, fidelity strengthened as the mean length of foraging bouts increased (guillemot: est = -0.25, SE = 0.14, *P* = 0.09; razorbill: est = -0.13, SE = 0.04, *P* = 0.001; Fig. S4) but decreased as the total time spent foraging increased (guillemot: est = 0.235, SE = 0.12, *P* = 0.05; razorbill: est = 0.07, SE = 0.04, *P* = 0.08; Fig. S5). The same patterns were true for the distance between distal points (Table S6; Fig. S4 & S5), but there was no significant relationship between NND and either the time to first foraging bout or the proportion of time spent foraging (Table S6).

When we used the difference in trip bearing as our fidelity metric, our results were similar for razorbills, with consecutive trips being more similar in their bearing, suggesting stronger fidelity, when the mean length of foraging bouts was higher (Est = -0.17, SE = 0.06, *P* = 0.003), but less similar when an individual spent more time foraging on the initial trip (Est = 0.09, SE = 0.05, *P* = 0.08). For guillemots, there was no clear evidence for any relationships between the difference in trip bearings and foraging metrics (Table S6).

### Characterising trip similarity between individuals over short timescales

In all species, conspecifics showed significantly greater similarity in their trip characteristics when their trips were made on the same day versus on different days. In guillemots, kittiwakes, and razorbills, this was consistent regardless of the fidelity metric used, but in puffins, we only found greater similarity between conspecifics foraging on the same day versus on different days when examining the distance between trip distal points (Table S7). In contrast, there was little evidence to suggest greater within-day similarity when comparing individuals of different species (Table S8).

**Fig. 5:**
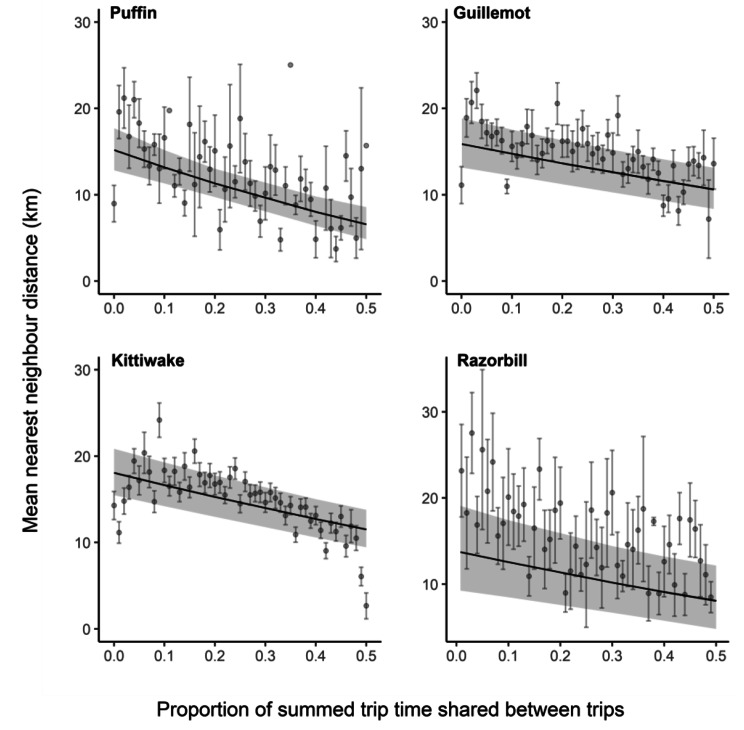
Relationship between the proportion of time that trips overlapped out at sea and the mean nearest neighbour distance between the trips. Shown are the raw data binned for clarity (means and standard errors) and model predictions with associated 95% confidence intervals

Puffins were the only species in which we found evidence that individuals that overlapped at the colony were more similar in their trip characteristics, with both NND and the distance between distal points being smaller when individuals had overlapped versus when they had not (NND: est = -0.46, SE = 0.16, *P* = 0.003; distal point distance: est = -0.51, SE = 0.19, *P* = 0.006; Fig. S6, Table S10). In contrast, overlapping in time when at sea was associated with greater similarity in trips as measured using NND and trip bearing in all species, though this relationship was stronger for puffins than for the other species (Fig. 5, Table S11). Results using the distance between distal points were similar, with greater overlap at sea associated with distal points that were significantly closer in space for puffins (est = -0.28, SE = 0.08, *P* = 0.001), kittiwakes (est = -0.24, SE = 0.03, *P* < 0.0001), and razorbills (est = -0.25, SE = 0.11, *P* = 0.02). In guillemots, the relationship trended in the same direction, but was not statistically significant (est = -0.05, SE = 0.05, *P* = 0.27).

## Discussion

Changes in movement behaviour are likely to shape critical responses to both slow and rapid changes in the environment for many species, including seabirds. However, certain behaviours, such as fidelity to specific routes or areas, may prove maladaptive if individuals continue to use areas that are declining in value or increasing in risk. Although fidelity is known to be commonplace throughout the animal kingdom, there remain considerable gaps in our knowledge of the mechanisms driving fidelity or indeed reducing it [16]. Pinpointing these processes will enhance understanding of how fidelity may change under environmental change and how such changes will affect population health. Here, we used between five and eight years of GPS data for four seabird species breeding at a single colony to characterise fidelity and provide new insights on the processes that may strengthen or weaken fidelity. All species displayed foraging site and route fidelity during chick rearing both within- and between-years indicating that individuals exhibit a tendency to use similar routes and/or return to similar foraging locations both over the short and longer term. The degree of fidelity between consecutive trips in guillemots and razorbills was also explained by consistent patterns in foraging behaviour, suggesting that individuals adjusted the similarity between their foraging trips according to our measures of foraging efficiency on the initial trip. Finally, we found evidence that individuals showed greater similarity in their trips when they overlapped to a greater degree in space and/or time, indicating the use of a common set of cues and/or information from con/heterospecifics when choosing their foraging route. This was particularly pronounced in puffins, where individuals showed greater similarity in their foraging routes if they overlapped temporally at the colony or out at sea with conspecifics.

Our finding of within-year foraging fidelity in each of the four species aligns with previous work in common guillemots and black-legged kittiwakes [22, 53–55]. Although all species displayed fidelity within a breeding season, the similarity between trips was generally higher for the auk species than kittiwakes, though this may be partly related to the larger foraging ranges of kittiwakes relative to the auk species on the Isle of May [36]. We also found evidence that fidelity extended beyond a single breeding season for common guillemots, black-legged kittiwakes, and razorbills, with the nearest neighbour distance being consistently lower within individuals than between individuals, even when comparing trips made in different years. This is despite previous work in this system demonstrating that species-specific foraging areas can vary markedly between years [36], suggesting that although individuals display fidelity even between years, there are marked differences between individuals of the same species in their foraging routes and areas. Indeed, we found evidence for significant between-individual variation in fidelity, particularly within years.

We used three different fidelity metrics to examine fidelity over subtly different aspects of trips. The nearest neighbour distance allowed us to characterise fidelity over the entire foraging trip, including the commutes to and from foraging grounds as well as the foraging locations themselves. In contrast, both the distance between distal points and the difference in trip bearing were focused on the likely terminal foraging location but allowed us to look at fidelity to this terminal location and fidelity in terms of travel direction from the colony respectively. For the within year fidelity analysis our results were largely consistent regardless of the metric we chose, suggesting that individuals tended to show fidelity both to the terminal foraging locations and over the routes used to reach them. However, our results did depend somewhat on the metric used when considering between year fidelity. In this case, when using the nearest neighbour distance, we found evidence for fidelity in each of the three species considered (kittiwake, guillemot, razorbill), but when using the distance between distal points and difference in trip bearing, the difference between within-individual and between-individual comparisons was only statistically significant for guillemots. This, in combination with the fact that mean NNDs were markedly smaller than the distance between distal points, may suggest that individuals were more faithful to the routes travelled than in a trip’s terminal location. However, we also suggest that the differences between species are likely to come from differences in data availability and thus statistical power, with 24 guillemots available for this analysis in contrast to 7 and 5 for kittiwakes and razorbills respectively. This is supported by the fact that effect sizes were largely similar between species.

The existence of fidelity to foraging areas and routes over both annual and inter-annual timescales likely points to there being benefits of fidelity to individuals within the Isle of May population. It potentially indicates predictable occurrence of prey within the broader foraging area, at least at a relatively coarse scale. Though the prey resources relied upon by seabirds were long assumed to be highly unpredictable, it is now considered that in northern and polar seas, prey occurrence is predictable at the scale of tens to hundreds of kilometres [25, 53]. Where the environment is predictable, fidelity is likely to reduce the time needed to find food and avoid the potential costs of initiating a new search each time [18]. Thus, it may be possible for individuals to reduce their movement costs by returning to areas where prey are reliably located. It is also possible that fidelity may confer other benefits. For example, it has been suggested that individual specialisations, such as fidelity, may be related to density with both theoretical and empirical work demonstrating that specialisation can be beneficial as density increases by shielding individuals from competition [56, 57]. Similarly, it has been suggested that fidelity may arise as a result of site familiarity, with the acquisition of detailed information about specific sites putting individuals at a competitive advantage in these locations [5, 23, 53]. Indeed, seabirds face substantial constraints when foraging in the breeding season, from incomplete knowledge of prey availability and competition with birds from other colonies to high energetic demands and pronounced limitations on how long they can be away from the nest. Thus, returning to familiar locations may prove to be markedly less risky than actively exploring new areas, promoting fidelity even where more profitable locations are available [23]. Uncovering if and how fidelity confers benefits within our system, and more broadly, requires further work. Indeed, few studies to date have provided evidence for a positive effect of foraging-site fidelity on foraging efficiency and reproductive success [58, 59].

Despite the potential benefits of fidelity, it is also possible for fidelity to drive individuals to make maladaptive foraging choices, particularly in the face of environmental change [9]. Seabirds are experiencing rapid human-induced environmental change across the globe, including climate change, fisheries competition, and the expansion of offshore renewable developments [60], and if individuals continue to prefer previously used routes or foraging areas, despite the fitness benefits of previously used areas being reduced by environmental change, then individuals may find themselves in a so-called ‘fidelity induced ecological trap’ [9]. Fidelity induced ecological traps have now been empirically demonstrated [11, 12, 61–63], and shown to lead to population-level consequences [11, 12], highlighting the importance of considering the role of fidelity in mediating individual and population responses to human-induced environmental change. However, fidelity to foraging areas or routes is rarely absolute (e.g., 14, 58). We found evidence that individuals may use information from their own foraging activities as well as from environmental cues and/or via observing the behaviour of others to determine their foraging sites/routes, indicating that despite displaying fidelity, individuals exhibit flexibility in their foraging locations/routes. Such flexibility may help to confer resilience to localised changes in the environment.

Both guillemots and razorbills adjusted the strength of their fidelity according to their foraging behaviour on a trip. Specifically, our results suggest that individuals tended to make a more similar trip following a trip where the total time spent foraging was lower (i.e., individuals found food relatively quickly across their trip) but also where their foraging was distributed over fewer, longer foraging bouts. This latter result suggests that, in line with our expectation, longer foraging bouts correspond to individuals exploiting high value patches for longer [46, 50, 64]. Combining these results with our finding that guillemots and razorbills show fidelity even between breeding seasons suggests that individuals may follow a hierarchical strategy, whereby they show long-term fidelity but use information on their foraging efficiency over single trips to fine tune their foraging trips. It also suggests that individuals may use relatively complex rules when selecting their foraging routes. For example, in this case, it was not only the total time spent foraging over a trip that predicted the similarity between consecutive trips, but also how the time spent foraging was distributed across the trip.

A fidelity strategy that allows the adjustment of fidelity in response to gathered information is likely to be associated with greater behavioural flexibility than a strategy where fidelity is absolute (i.e., ‘always-stay’) and is therefore expected to facilitate responses of site faithful species to environmental change [9]. Such flexibility allows individuals to adjust their behaviour in response to information gained by themselves or others, and in so doing reduce the strength of fidelity over time [9]. Thus, the fact that guillemots and razorbills appear to moderate their fidelity based on recent information on foraging success may allow them to alter their movement in response to changes in the value or risk of the marine environment. For example, if human activity leads to a reduction in food availability in an area frequently used by an individual, their probability of returning to this local area will be expected to decline over time, thereby potentially offsetting negative effects of the human-induced change on fitness. Such responses have been demonstrated empirically in a small number of cases. For example, a study of little penguins (*Eudyptula minor*) demonstrated that individuals showed higher fidelity after foraging trips when prey capture success was high, and that this strategy was beneficial for foraging success [65]. Similarly, a study in bison (*Bison bison*) demonstrated that although individuals show fidelity to a specific set of meadows, a reduction in forage quality leads to a reduction in fidelity [15]. Though such a strategy may prove advantageous in the face of environmental change, its effectiveness is likely to depend on the reliability of cues or of previous experience [7, 66]. For example, in red-necked grebes (*Podiceps grisegena*) the reliance of individuals on information gained in the previous breeding season resulted in the strategy being maladaptive due to unpredictable changes in food availability between years as a result of human activity [63]. Furthermore, such flexibility may prove inadequate in facilitating responses to large-scale environmental changes (e.g., climate change) when species exhibit very strong breeding site fidelity. This is because environmental change acting over very large spatial scales, such as climate change, will likely lead to the devaluation of foraging habitat over time, meaning that both preferred and avoided sites/routes are likely to be devalued to similar extents. Thus, when individuals continue to return to the same breeding site over time regardless of the value of that site to fitness due to strong fidelity, smaller adjustments in foraging behaviour are unlikely to prove a sufficient response.

We also found additional evidence that individuals display flexibility in their choice of foraging site or route. Conspecifics showed greater similarity in their trips when they overlapped to a greater degree in space and/or time, suggesting that they adjust their movement decisions based on information gained locally at the time of making a trip. Specifically, for all species we found that individuals foraging on the same day were more similar in their trips than those foraging on different days, and that individuals whose trips overlapped in time to a greater degree were more similar in their trips. We suggest that these results may indicate that individuals are using the same indicators of prey distribution and availability, such as olfactory [67] and visual cues [68], when selecting their foraging areas or routes and/or that they use information on the presence of other individuals when choosing where to go (e.g., conspecific attraction/local enhancement) [19, 49, 69]. The tendency for colonial seabirds to be attracted to other foraging conspecifics has been demonstrated on multiple occasions [19, 69, 70], and such behaviour may be beneficial to individuals when responding to environmental change if the behaviour of others is a good indication of habitat quality. However, a combination of fidelity and conspecific attraction could create a perceptual trap, whereby individuals choose locations that are not the best available [71].

We also found that puffins that overlapped at the colony were more similar in their foraging trips than those that did not. This may again suggest that individuals carrying out foraging trips over a similar time period respond to the same local environmental cues, but it could also suggest that individuals use information gained from observing or interacting with others (i.e., social information) when deciding on their foraging routes. There is some existing evidence that seabirds use social information to determine their foraging locations, including that individuals respond to the flight directions of conspecifics [49, 72] and/or to the prey brought back by them [72] when choosing where to forage. For example, Australasian gannets (*Morus serrator*) that overlapped at the colony were more similar in the location of their initial foraging patches than those that did not overlap prior to departure [73]. Nevertheless, we cannot be sure of the precise mechanism underpinning our finding that overlap at the colony was linked to higher trip similarity in puffins, and thus additional work will be needed to pinpoint the drivers of this result. However, given the potential importance that social information transfer may play in driving movement patterns, including the prevalence and magnitude of fidelity, we call for further work examining the interactions of individuals of the same and different species when foraging at sea and relating such interactions to subsequent foraging decisions and/or success. This may be facilitated by the continued development of bio-logging technologies which enable remote recording of individual interactions [74].

## Conclusions

In summary, our results suggest that fidelity to foraging routes and areas both within and between breeding seasons is present in common guillemots, Atlantic puffins, black-legged kittiwakes, and razorbills breeding on the Isle of May. Such fidelity is likely to prove beneficial if resources are predictable or if it provides individuals with a competitive advantage, but could prove costly in the face of continuing human-induced environmental change by driving individuals to continue using areas being degraded by human activity. Further work examining the relationships between fidelity and metrics of success, such as diet and reproductive success, particularly over periods where environmental conditions are known to have changed will be instrumental for understanding the potential costs of fidelity under continued environmental change. Nevertheless, our findings that fidelity was predicted by foraging success in guillemots and razorbills, and by the foraging routes of conspecifics, suggests that individuals may be able to use information gained while foraging to reduce the intensity of fidelity and adjust to changing conditions. The degree to which these mechanisms will facilitate resilience will depend on an array of factors, highlighting the challenge of understanding what fidelity means for species in a rapidly changing world. Given the multitude of potential outcomes of fidelity in the face of anthropogenic pressures, more work will be needed to both understand how different sources of environmental change modify the quality and predictability of habitats, as well as the extent to which organisms can integrate cues, prior experience, and information gained from others to make informed movement decisions. Such understanding will make it possible to better integrate fidelity into predictions of the impacts of environmental change on populations and into actions to reduce or compensate for such impacts.

### Electronic supplementary material

Below is the link to the electronic supplementary material.

Supplementary Material 1

## Data Availability

The data needed to replicate the analyses presented in this paper are available at https://doi.org/10.6084/m9.figshare.25637175.v1

## Abbreviation

NND: Nearest neighbour distance
