# Supplementary material for: Seabirds show foraging site and route fidelity but demonstrate flexibility in response to local information
Source: Mov Ecol. 2024 Jun 13;12:46. doi: 10.1186/s40462-024-00467-9 (PMC11177427; doi:10.1186/s40462-024-00467-9)
Supplement: Supplementary file 1 — Supplementary Material 1 [file 40462_2024_467_MOESM1_ESM.docx]

**Supporting Information**

**Seabirds show foraging site and route fidelity but demonstrate flexibility in response to local information**


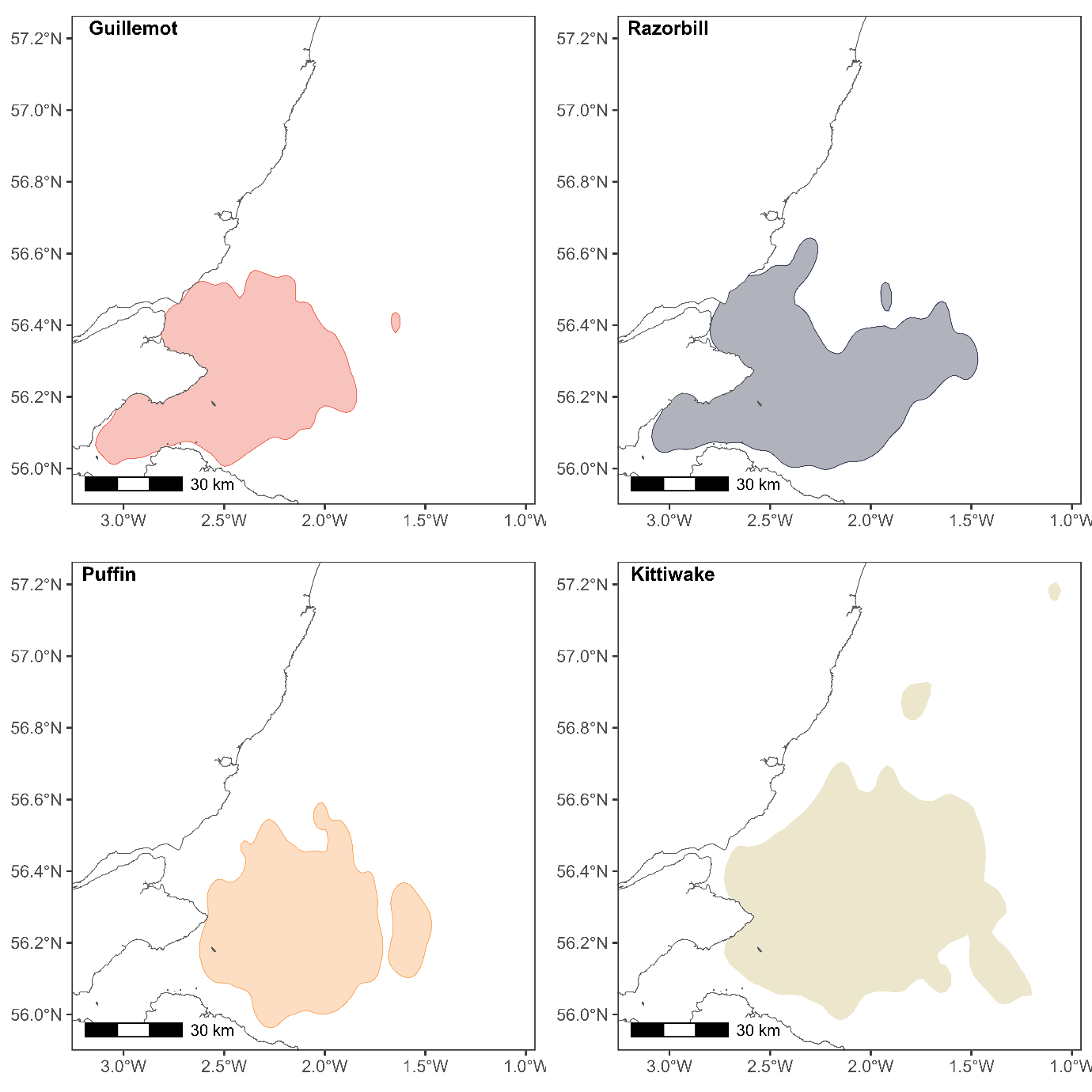
Charlotte. E. Regan, Maria. I. Bogdanova, Mark Newell, Carrie Gunn, Sarah Wanless, Mike. P. Harris, Samuel Langlois Lopez, Ella Benninghaus, Francis Daunt & Kate. R. Searle

**Figure S1.** Foraging ranges for guillemots, razorbills, puffins and kittiwakes breeding on the Isle of May. Shown are 90% utilisation distribution kernel contours calculated using adehabitatHR on at sea locations for individuals tracked in all years between 2010 and 2021.


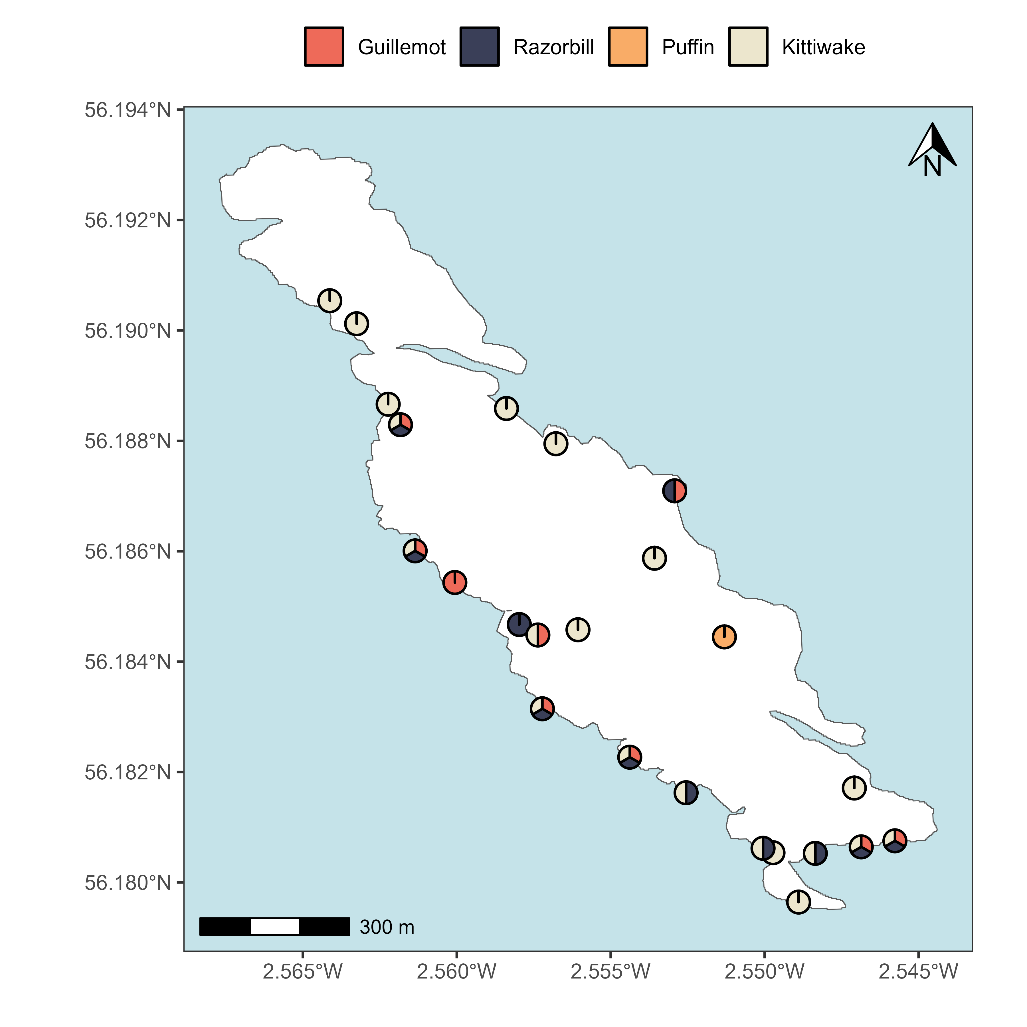


**Figure S2.** Capture areas used for GPS tagging on the Isle of May, with colours illustrating the species captured at each site

**Table S1.** Details on the tagging protocol for birds of the four species tagged on the Isle of May, whose data were used in the associated analyses.

| **Year** | **Species** | **Capture areas** | **# birds** |  | **Capture method** | **Tag type*** | **Weight & dimensions** | **Attachment method** | **Fix schedule** | **Tracking duration (days)** |  | **Tracking period** |
| --- | --- | --- | --- | --- | --- | --- | --- | --- | --- | --- | --- | --- |
| 2010 | Guillemot | 3 | 33 |  | Noose pole | IgotU | 15.0g; 40x22x11 | Taped to back feathers | 1 min | 0.94 – 2.83 |  | 10^th^ June - 18^th^ June |
|  | Kittiwake | 10 | 33 |  | Noose pole | IgotU/GiPSy | 15.0g/11.5g;  40x22x11/48x20x9 | Taped to back feathers | 1 min/1 min on 5 min off | 0.66 – 2.76 |  | 29^th^ May – 27^th^ June |
|  | Razorbill | 6 | 17 |  | Noose pole | IgotU | 15.0g; 40x22x11 | Taped to back feathers | 1 min | 0.38 – 2.79 |  | 8^th^ June – 18^th^ June |
|  | Puffin | 1 | 4 |  | In burrow | IgotU/GiPSy | 15.0g/11.5g;  40x22x11/48x20x9 | Taped to back feathers | 1 min/1 min on 5 min off | 1.04 – 2.46 |  | 19^th^ June – 25^th^ June |
| 2012 | Guillemot | 5 | 17 |  | Noose pole | IgotU | 17.1g; 40x22x11 | Taped to back feathers | 2 min | 0.60 – 3.86 |  | 11^th^ June – 19^th^ June |
|  | Kittiwake | 6 | 17 |  | Noose pole | IgotU | 14.2g; 40x22x11 | Taped to back feathers | 2 min | 0.94 – 2.27 |  | 29^th^ May – 9^th^ June |
|  | Razorbill | 8 | 13 |  | Noose pole | IgotU | 17.7g; 40x22x11 | Taped to back feathers | 2 min | 0.62 – 3.01 |  | 11^th^ June – 21^st^ June |
|  | Puffin | 1 | 0 |  | - | - | - | - | - | - |  | - |
| 2013 | Guillemot | 5 | 20 |  | Noose pole | IgotU | 17.8g; 40x22x11 | Taped to back feathers | 2 min | 1.19 – 3.63 |  | 26^th^ June – 2^nd^ July |
|  | Kittiwake | 9 | 21 |  | Noose pole | IgotU | 16.4g; 40x22x11 | Taped to back feathers | 2 min | 0.76 – 2.97 |  | 24^th^ June – 15^th^ July |
|  | Razorbill | 3 | 7 |  | Noose pole | IgotU | 17.7g; 40x22x11 | Taped to back feathers | 2 min | 2.52 – 4.95 |  | 26^th^ June – 2^nd^ July |
|  | Puffin | 1 | 0 |  | - | - | - | - | - | - |  | - |
| 2014 | Guillemot | 4 | 8 |  | Noose pole | IgotU | 17.5g; 40x22x11 | Taped to back feathers | 2 min | 1.87 – 2.84 |  | 22^nd^ June – 27^th^ June |
|  | Kittiwake | 3 | 11 |  | Noose pole | IgotU | 15.3g; 40x22x11 | Taped to back feathers | 2 min | 0.98 – 2.34 |  | 29^th^ June – 8^th^ July |
|  | Razorbill | 2 | 5 |  | Noose pole | IgotU | 17.7g; 40x22x11 | Taped to back feathers | 2 min | 2.38 – 2.93 |  | 18^th^ June – 21^st^ June |
|  | Puffin | 1 | 0 |  | - | - | - | - | - | - |  | - |
| 2018 | Guillemot | 3 | 24 |  | Noose pole | Pathtrack | 11.0g; 50x23x10 | Taped to back feathers | 5 min | 0.54 – 9.98 |  | 26^th^ June – 7^th^ July |
|  | Kittiwake | 3 | 16 |  | Noose pole | Pathtrack | 4.1g; 40x12x10 | Taped to back feathers | 5 min | 1.77 – 4.61 |  | 21^st^ June – 2^nd^ July |
|  | Razorbill | 2 | 13 |  | Noose pole | Pathtrack | 8.2g; 50x13x10 | Taped to back feathers | 10 min | 0.33 – 9.68 |  | 26^th^ June – 6^th^ July |
|  | Puffin | 1 | 17 |  | Mist nets | Pathtrack | 8.2g/4.1g;  50x13x10/40x12x10 | Taped to back feathers | 10 min | 1.65 – 15.34 |  | 20^th^ June – 25^th^ July |
| 2019 | Guillemot | 3 | 23 |  | Noose pole | Pathtrack | 11.0g; 51x24x9 | Taped to back feathers | 5 min | 0.51 – 6.73 |  | 21^st^ June – 4^th^ July |
|  | Kittiwake | 2 | 25 |  | Noose pole | Pathtrack | 4.1g; 42x14x8 | Taped to tail feathers | 5 min/10 min | 0.94 – 9.92 |  | 18^th^ June – 3^rd^ July |
|  | Razorbill | 2 | 13 |  | Noose pole | Pathtrack | 8.2g; 50x15x10 | Taped to back feathers | 10 min | 4.57 – 9.03 |  | 21^st^ June – 30^th^ June |
|  | Puffin | 1 | 23 |  | Purse nets at burrow | Pathtrack | 3.2g; 42x14x8 | Taped to back feathers | 10 min | 1.43 – 5.48 |  | 29^th^ June – 10^th^ July |
| 2020 | Guillemot | 3 | 21 |  | Noose pole | Pathtrack GPS+TDR | 16.5g; 59x24x11 | Taped to back feathers | 5 min | 0.28 – 11.44 |  | 23^rd^ June – 6^th^ July |
|  | Kittiwake | 2 | 22 |  | Noose pole | UvA-BiTS | 9.0g; 62x30x12 | Glued to back feathers | 10 sec or 64 sec in wind farm footprint, 5 min elsewhere at sea, 15 min at colony | 5.40 – 36.75 |  | 27^th^ June – 7^th^ August |
|  | Razorbill | 2 | 13 |  | Noose pole | Pathtrack GPS+TDR | 13.0g; 54x24x10 | Taped to back feathers | 5 min | 0.26 – 12.90 |  | 23^rd^ June – 6^th^ July |
|  | Puffin | 1 | 7 |  | Purse nets at burrow | Pathtrack | 3.4g; 42x14x8 | Taped to back feathers | 10 min | 0.04 – 8.40 |  | 29^th^ June – 11^th^ July |
| 2021 | Guillemot | 2 | 23 |  | Noose pole | Pathtrack GPS+TDR | 16.5g; 59x24x11 | Taped to back feathers | 5 min | 0.85 – 10.09 |  | 21^st^ June – 1^st^ July |
|  | Kittiwake | 4 | 50 |  | Noose pole | Pathtrack/UvA-BiTS | 4.0g/9.0g;  42x14x8/62x30x12 | Taped to tail feathers/ glued to back feathers | 5 min/64 sec in wind farm footprint, 5 min elsewhere at sea, 15 min at colony | 3.42 – 26.27 |  | 28^th^ June – 27^th^ July |
|  | Razorbill | 4 | 11 |  | Noose pole | Pathtrack GPS+TDR | 12.8g; 54x24x10 | Taped to back feathers | 5 min | 2.13 – 10.25 |  | 21^st^ June – 1^st^ July |
|  | Puffin | 1 | 24 |  | Purse nets at burrow | Pathtrack | 3.4g; 42x14x8 | Taped to back feathers | 10 min | 2.14 – 9.33 |  | 2^nd^ July – 13^th^ July |

* Tag models used: IgotU GT-120; GiPSy-2 and GiPSy-3; Pathtrack nanoFix-GEO

**Table 2.** Summary of data used for the between-year fidelity analyses.

| **Species** | **Number of individuals** | **Years** | **Years between comparison years**  **Median (range)** |
| --- | --- | --- | --- |
| Guillemot | 24 | 2010, 2012, 2013, 2014, 2018, 2019, 2020, 2021 | 4.5 (1 – 9) |
| Razorbill | 5 | 2010, 2012, 2013, 2014, 2018, 2019, 2020, 2021 | 2 (2 – 4) |
| Kittiwake | 7 | 2012, 2013, 2018, 2019, 2020, 2021 | 1.5 (1 – 3) |

**Determining flight speed cut-offs**

We used data on observed movement speeds to determine a cut-off to exclude improbable movements. In each case, we selected 30 m/s as for each species, movements above this threshold were very rare.


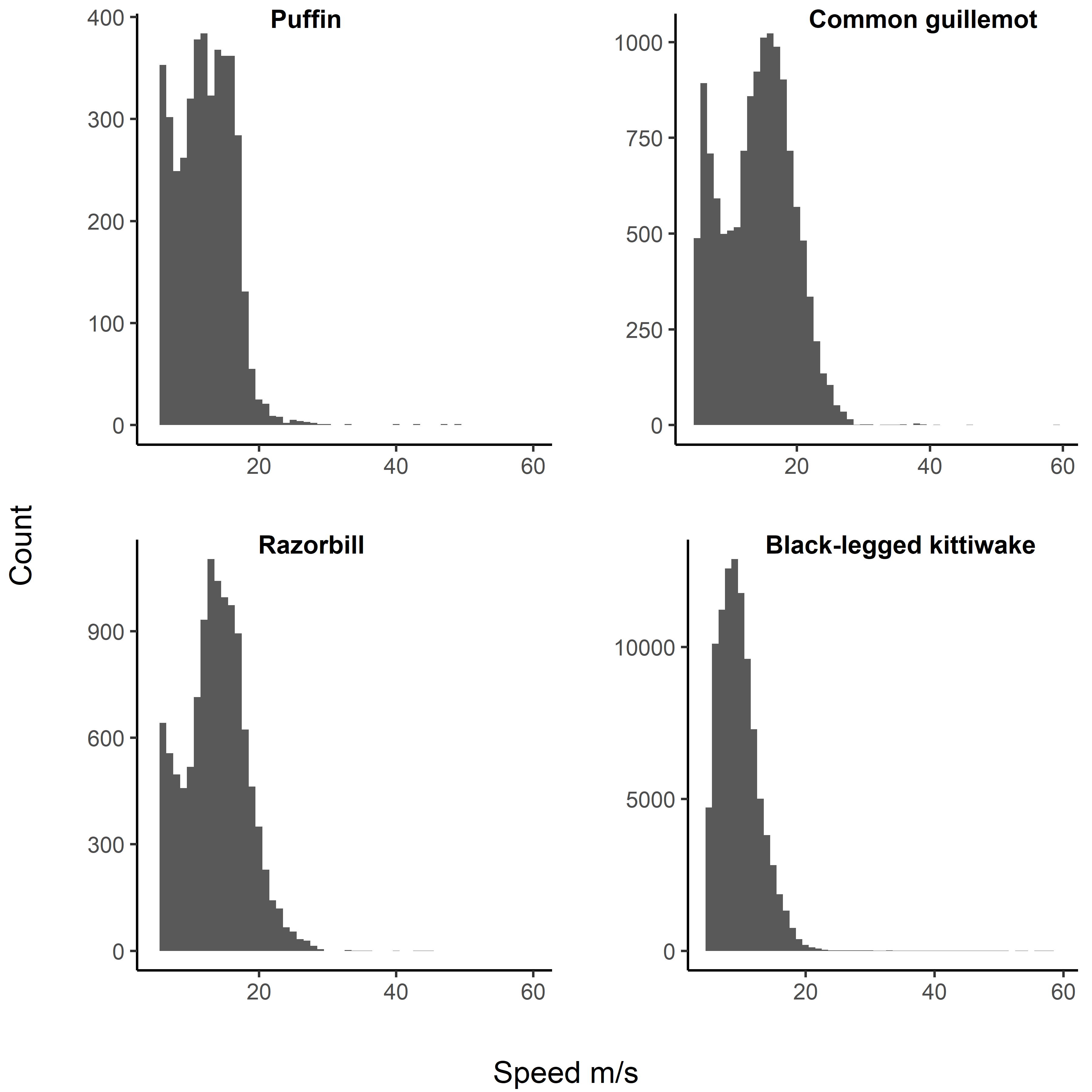


**Figure S3.** Histograms of individual movement speeds across the tracking period. Speeds less than 5 metres per second and greater than 60 metres per second were excluded to aid clarity.

**Within year fidelity model results**

**Table S3.** Model estimates, standard errors, and P values, from models of within-year fidelity including main effect terms only.

|  | **Guillemot** | | | **Kittiwake** | | | **Razorbill** | | | **Puffin** | | | | |
| --- | --- | --- | --- | --- | --- | --- | --- | --- | --- | --- | --- | --- | --- | --- |
|  | Est | SE | P | Est | SE | P | Est | SE | P | Est | SE | | P |  |
| **Nearest neighbour distance** | | | | | | | | | |  | | | | |
| Intercept | 3.932 | 0.140 | <0.001 | 3.976 | 0.091 | <0.001 | 3.887 | 0.234 | <0.001 | 4.074 | | 0.141 | 0.002 | |
| Comparison type (within individual) | -0.827 | 0.049 | <0.001 | -0.426 | 0.018 | <0.001 | -0.564 | 0.045 | <0.001 | -0.675 | | 0.065 | <0.001 | |
| Time difference | 0.200 | 0.046 | <0.001 | 0.008 | 0.009 | 0.391 | -0.011 | 0.030 | 0.697 | 0.007 | | 0.048 | 0.892 | |
| Distance between capture sites | 0.038 | 0.022 | 0.085 | 0.076 | 0.007 | <0.001 | 0.312 | 0.052 | <0.001 |  | |  |  | |
|  | Var | SD | P | Var | SD | P | Var | SD | P | Var | | SD | P | |
| Year | 0.131 | 0.363 | <0.001 | 0.042 | 0.205 | <0.001 | 0.405 | 0.637 | <0.001 | 0.062 | | 0.249 | 0.014 | |
| Loggers:Year | 0.094 | 0.307 | <0.001 | 0.088 | 0.297 | <0.001 | 0.122 | 0.349 | <0.001 | 0.060 | | 0.245 | <0.001 | |
| **Difference between distal points** | | | | | | | | | |  | | | | |
| Intercept | 5.290 | 0.191 | <0.001 | 5.814 | 0.117 | <0.001 | 5.421 | 0.286 | <0.001 | 5.120 | | 0.072 | <0.001 | |
| Comparison type (within individual) | -1.123 | 0.063 | <0.001 | -0.610 | 0.026 | <0.001 | -0.748 | 0.063 | <0.001 | -0.790 | | 0.071 | <0.001 | |
| Time difference | 0.190 | 0.058 | 0.001 | 0.098 | 0.011 | <0.001 | 0.473 | 0.073 | <0.001 | 0.052 | | 0.052 | 0.321 | |
| Distance between capture sites | 0.066 | 0.028 | 0.019 | -0.004 | 0.013 | 0.746 | -0.059 | 0.041 | 0.152 |  | | | | |
|  | Var | SD | P | Var | SD | P | Var | SD | P | Var | SD | | P | |
| Year | 0.245 | 0.495 | <0.001 | 0.059 | 0.242 | <0.001 | 0.593 | 0.770 | <0.001 | 0.002 | 0.039 | | 0.89 | |
| Loggers:Year | 0.214 | 0.462 | <0.001 | 0.231 | 0.481 | <0.001 | 0.226 | 0.475 | <0.001 | 0.104 | 0.323 | | <0.001 | |
| **Difference in bearing** | | | | | | | | | |  | | | | |
| Intercept | 1.179 | 0.042 | <0.001 | 0.878 | 0.064 | <0.001 | 1.213 | 0.032 | <0.001 | 0.854 | 0.024 | | <0.001 | |
| Comparison type (within individual) | -0.197 | 0.016 | <0.001 | -0.070 | 0.004 | <0.001 | -0.109 | 0.017 | <0.001 | -0.017 | 0.017 | | 0.326 | |
| Time difference | 0.028 | 0.019 | 0.153 | 0.011 | 0.002 | <0.001 | 0.121 | 0.023 | <0.001 | 0.017 | 0.013 | | 0.193 | |
|  | Var | SD | P | Var | SD | P | Var | SD | P | Var | SD | | P | |
| Year | 0.008 | 0.091 | 0.013 | 0.027 | 0.165 | <0.001 | 0.002 | 0.042 | 0.625 | 0.000 | 0.000 | | 1 | |
| Loggers:Year | 0.043 | 0.208 | <0.001 | 0.027 | 0.166 | <0.001 | 0.030 | 0.174 | <0.001 | 0.022 | 0.148 | | <0.001 | |

**Table S4.** Model estimates, standard errors, and P values, from models of within-year fidelity including a first order interaction between the comparison type (within or between individual) and the time between trip starts.

|  | **Guillemot** | | | **Kittiwake** | | | **Razorbill** | | | **Puffin** | | | | |
| --- | --- | --- | --- | --- | --- | --- | --- | --- | --- | --- | --- | --- | --- | --- |
|  | Est | SE | P | Est | SE | P | Est | SE | P | Est | SE | | P |  |
| **Nearest neighbour distance** | | | | | | | | | |  | | | | |
| Intercept | 3.903 | 0.140 | <0.001 | 4.005 | 0.092 | <0.001 | 3.822 | 0.232 | <0.001 | 4.089 | | 0.151 | 0.002 | |
| Comparison type (within individual) | -0.672 | 0.067 | <0.001 | -4.533 | 0.018 | <0.001 | -0.366 | 0.068 | <0.001 | -0.461 | | 0.104 | <0.001 | |
| Time difference | 0.097 | 0.055 | 0.078 | 0.033 | 0.009 | <0.001 | 0.152 | 0.067 | 0.022 | -0.030 | | 0.050 | 0.545 | |
| Distance between capture sites | 0.039 | 0.022 | 0.076 | 0.002 | 0.009 | 0.857 | -0.026 | 0.030 | 0.384 |  | |  |  | |
| Comparison type*time difference | 0.305 | 0.089 | <0.001 | 0.099 | 0.014 | <0.001 | 0.381 | 0.098 | <0.001 | 0.363 | | 0.139 | 0.009 | |
|  | Var | SD | P | Var | SD | P | Var | SD | P | Var | | SD | P | |
| Year | 0.129 | 0.360 | <0.001 | 0.044 | 0.209 | <0.001 | 0.394 | 0.628 | <0.001 | 0.075 | | 0.274 | 0.008 | |
| Identity:Year | 0.092 | 0.304 | <0.001 | 0.090 | 0.300 | <0.001 | 0.119 | 0.345 | <0.001 | 0.059 | | 0.243 | <0.001 | |
| **Difference between distal points** | | | | | | | | | |  | | | | |
| Intercept | 5.249 | 0.190 | <0.001 | 5.867 | 0.120 | <0.001 | 5.316 | 0.284 | <0.001 | 5.129 | | 0.080 | <0.001 | |
| Comparison type (within individual) | -0.910 | 0.085 | <0.001 | -0.658 | 0.027 | <0.001 | -0.430 | 0.095 | <0.001 | -0.509 | | 0.115 | <0.001 | |
| Time difference | 0.049 | 0.070 | 0.485 | 0.021 | 0.014 | 0.127 | 0.217 | 0.092 | 0.019 | 0.005 | | 0.055 | 0.933 | |
| Distance between capture sites | 0.068 | 0.028 | 0.016 | -0.016 | 0.013 | 0.247 | -0.082 | 0.041 | 0.048 |  | | | | |
| Comparison type*time difference | 0.418 | 0.114 | <0.001 | 0.177 | 0.020 | <0.001 | 0.611 | 0.136 | <0.001 | 0.475 | 0.153 | | 0.002 | |
|  | Var | SD | P | Var | SD | P | Var | SD | P | Var | SD | | P | |
| Year | 0.241 | 0.491 | <0.001 | 0.062 | 0.248 | <0.001 | 0.577 | 0.759 | <0.001 | 0.006 | 0.075 | | 0.650 | |
| Identity:Year | 0.209 | 0.457 | <0.001 | 0.237 | 0.487 | <0.001 | 0.219 | 0.468 | <0.001 | 0.101 | 0.318 | | <0.001 | |
| **Difference in bearing** | | | | | | | | | |  | | | | |
| Intercept | 1.174 | 0.042 | <0.001 | 0.884 | <0.001 | 1.165 | 0.037 | <0.001 |  | 0.854 | 0.024 | | <0.001 | |
| Comparison type (within individual) | -0.169 | 0.025 | <0.001 | -0.075 | <0.001 | 0.024 | 0.029 | 0.410 |  | -0.026 | 0.028 | | 0.339 | |
| Time difference | 0.008 | 0.023 | 0.722 | 0.001 | 0.678 | 0.019 | 0.030 | 0.513 |  | 0.018 | 0.013 | | 0.169 | |
| Comparison type*time difference | 0.058 | 0.038 | 0.127 | 0.023 | <0.001 | 0.242 | 0.044 | <0.001 |  | -0.016 | 0.037 | | 0.653 | |
|  | Var | SD | P | Var | SD | P | Var | SD | P | Var | SD | | P | |
| Year | 0.008 | 0.091 | 0.013 | 0.027 | 0.165 | <0.001 | 0.003 | 0.060 | 0.371 | 0.000 | 0.000 | | 1 | |
| Identity:Year | 0.043 | 0.208 | <0.001 | 0.028 | 0.166 | <0.001 | 0.030 | 0.172 | <0.001 | 0.022 | 0.148 | | <0.001 | |

**Between year fidelity model results**

**Table S5.** Model estimates, standard errors, and P values, from models of between-year fidelity including main effect terms only.

|  | **Guillemot** | | | **Kittiwake** | | | **Razorbill** | | |
| --- | --- | --- | --- | --- | --- | --- | --- | --- | --- |
|  | Est | SE | P | Est | SE | P | Est | SE | P |
| **Nearest neighbour distance** | | | | | | | | | |
| Intercept | 4.144 | 0.126 | <0.001 | 3.436 | 0.863 | 0.006 | 3.860 | 0.384 | <0.001 |
| Comparison type (within individual) | -1.225 | 0.174 | <0.001 | -1.324 | 0.438 | 0.005 | -1.201 | 0.299 | <0.001 |
| Time difference | 0.140 | 0.137 | 0.310 | 0.404 | 0.229 | 0.087 | 0.164 | 0.241 | 0.497 |
| Year difference | 0.006 | 0.056 | 0.916 | 0.921 | 1.506 | 0.600 | 0.224 | 0.516 | 0.666 |
| Distance between capture sites | 0.016 | 0.079 | 0.843 | -0.129 | 0.315 | 0.686 | -0.284 | 0.151 | 0.063 |
| **Difference between distal points** | | | | | | | | | |
| Intercept | 5.583 | 0.210 | <0.001 | 5.741 | 0.872 | <0.001 | 5.040 | 0.575 | <0.001 |
| Comparison type (within individual) | -0.984 | 0.205 | <0.001 | -0.877 | 0.605 | 0.159 | -0.392 | 0.347 | 0.261 |
| Time difference | 0.022 | 0.169 | 0.896 | -0.050 | 0.291 | 0.866 | -0.086 | 0.283 | 0.761 |
| Year difference | 0.046 | 0.090 | 0.610 | 1.658 | 1.050 | 0.338 | 0.394 | 0.621 | 0.528 |
| Distance between capture sites | -0.038 | 0.096 | 0.693 | -0.508 | 0.428 | 0.244 | -0.231 | 0.177 | 0.194 |
| **Difference in bearing** | | | | | | | | | |
| Intercept | 1.158 | 0.070 | <0.001 | 0.606 | 0.208 | 0.010 | 1.194 | 0.132 | <0.001 |
| Comparison type (within individual) | -0.226 | 0.067 | <0.001 | -0.124 | 0.128 | 0.342 | -0.092 | 0.108 | 0.396 |
| Time difference | -0.034 | 0.055 | 0.537 | 0.067 | 0.065 | 0.311 | -0.069 | 0.086 | 0.427 |
| Year difference | 0.033 | 0.030 | 0.276 | -0.038 | 0.297 | 0.905 | -0.030 | 0.184 | 0.871 |
| Distance between capture sites | -0.011 | 0.031 | 0.735 | 3.28 × 10^-5^ | 0.092 | 0.100 | 0.011 | 0.054 | 0.833 |

**Foraging behaviour model results**

**Table S6**. Model estimates, standard errors, and P values, from models examining relationships between the fidelity shown over consecutive trips and measures of foraging behaviour on the initial trip.

|  | **Guillemot** | | | **Razorbill** | | |
| --- | --- | --- | --- | --- | --- | --- |
|  | Est | SE | P | Est | SE | P |
| **Nearest neighbour distance** | | | | | | |
| Intercept | 3.328 | 0.219 | <0.001 | 1.899 | 0.091 | <0.001 |
| Time difference | 0.097 | 0.113 | 0.392 | 0.091 | 0.034 | 0.009 |
| Proportion time foraging | 0.107 | 0.152 | 0.481 | 0.003 | 0.041 | 0.942 |
| Time to first foraging bout | -0.015 | 0.117 | 0.898 | -0.019 | 0.034 | 0.576 |
| Mean foraging bout duration | -0.249 | 0.144 | 0.086 | -0.131 | 0.040 | 0.001 |
| Total foraging time | 0.235 | 0.119 | 0.049 | 0.065 | 0.037 | 0.081 |
| Year (2021) | -0.405 | 0.296 | 0.181 | -0.203 | 0.128 | 0.130 |
|  | Var | SD | P | Var | SD | P |
| Identity | 0.263 | 0.513 | 0.013 | 0.055 | 0.236 | <0.001 |
| **Difference between distal points** | | | | | | |
| Intercept | 4.482 | 0.251 | <0.001 | 5.225 | 0.413 | <0.001 |
| Time difference | 0.100 | 0.128 | 0.433 | 0.531 | 0.172 | 0.002 |
| Proportion time foraging | -0.015 | 0.172 | 0.929 | 0.047 | 0.208 | 0.823 |
| Time to first foraging bout | -0.054 | 0.132 | 0.684 | -0.099 | 0.174 | 0.568 |
| Mean foraging bout duration | -0.170 | 0.163 | 0.299 | -0.626 | 0.203 | 0.002 |
| Total foraging time | 0.318 | 0.135 | 0.019 | 0.455 | 0.185 | 0.015 |
| Year (2021) | -0.578 | 0.339 | 0.097 | -0.997 | 0.580 | 0.102 |
|  | Var | SD | P | Var | SD | P |
| Identity | 0.358 | 0.598 | 0.012 | 0.100 | 0.999 | <0.001 |
| **Difference in bearing** | | | | | | |
| Intercept | 1.043 | 0.087 | <0.001 | 0.857 | 0.095 | <0.001 |
| Time difference | -0.028 | 0.041 | 0.492 | 0.190 | 0.049 | <0.001 |
| Proportion time foraging | -0.010 | 0.055 | 0.855 | 0.087 | 0.060 | 0.145 |
| Time to first foraging bout | -0.004 | 0.043 | 0.929 | -0.055 | 0.050 | 0.276 |
| Mean foraging bout duration | 0.038 | 0.053 | 0.472 | -0.171 | 0.057 | 0.003 |
| Total foraging time | -0.024 | 0.043 | 0.587 | 0.093 | 0.052 | 0.079 |
| Year (2021) | -0.188 | 0.118 | 0.123 | 0.109 | 0.132 | 0.418 |
|  | Var | SD | P | Var | SD | P |
| Identity | 0.055 | 0.234 | 0.005 | 0.031 | 0.175 | 0.061 |


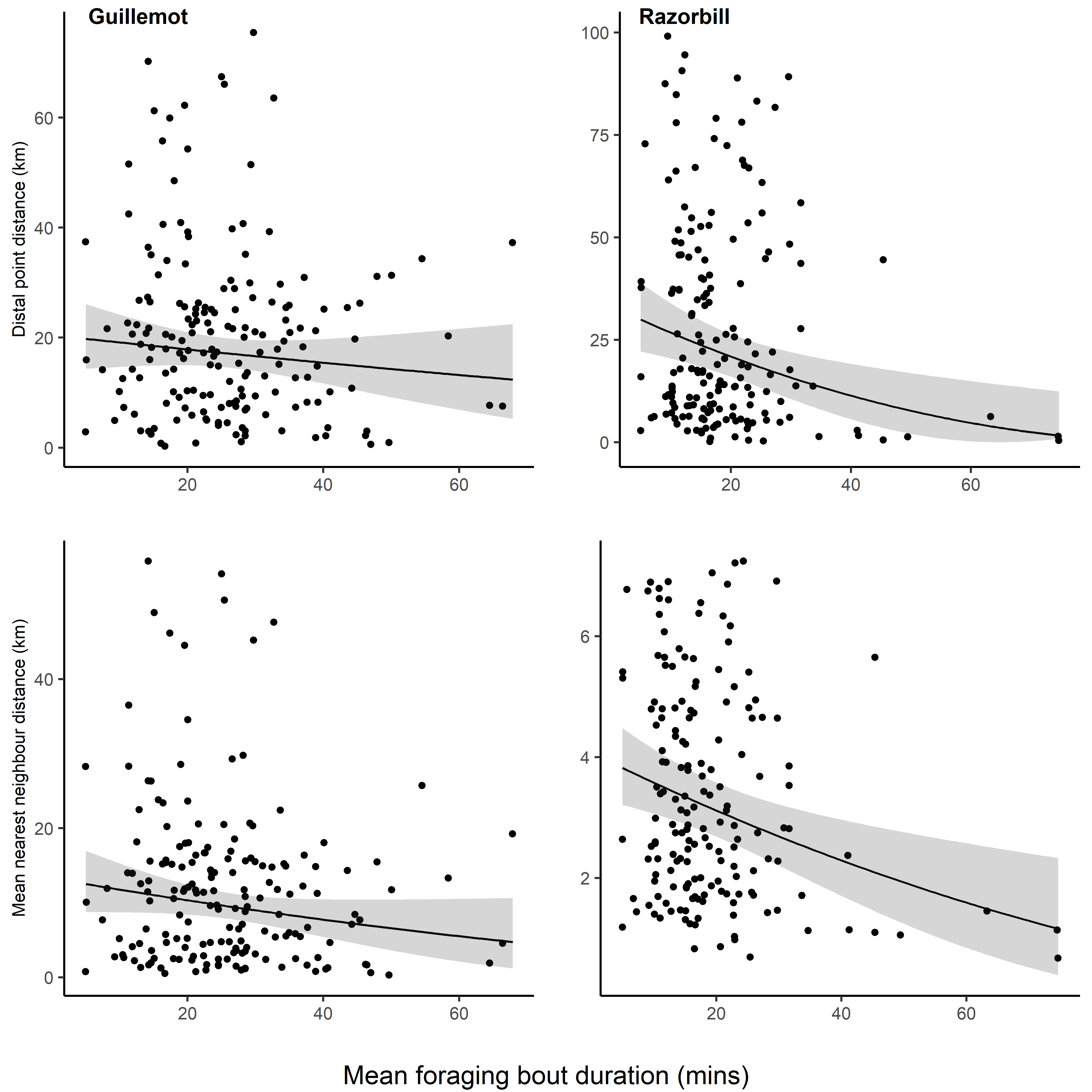


**Figure S4.** Relationship between the mean length of foraging bouts on the initial trip and the distance between the distal point of the initial trip and the subsequent trip made by an individual (top row) and the mean nearest neighbour distance between consecutive trips (bottom row). Shown are the raw data (points) and model predictions with associated 95% confidence intervals.


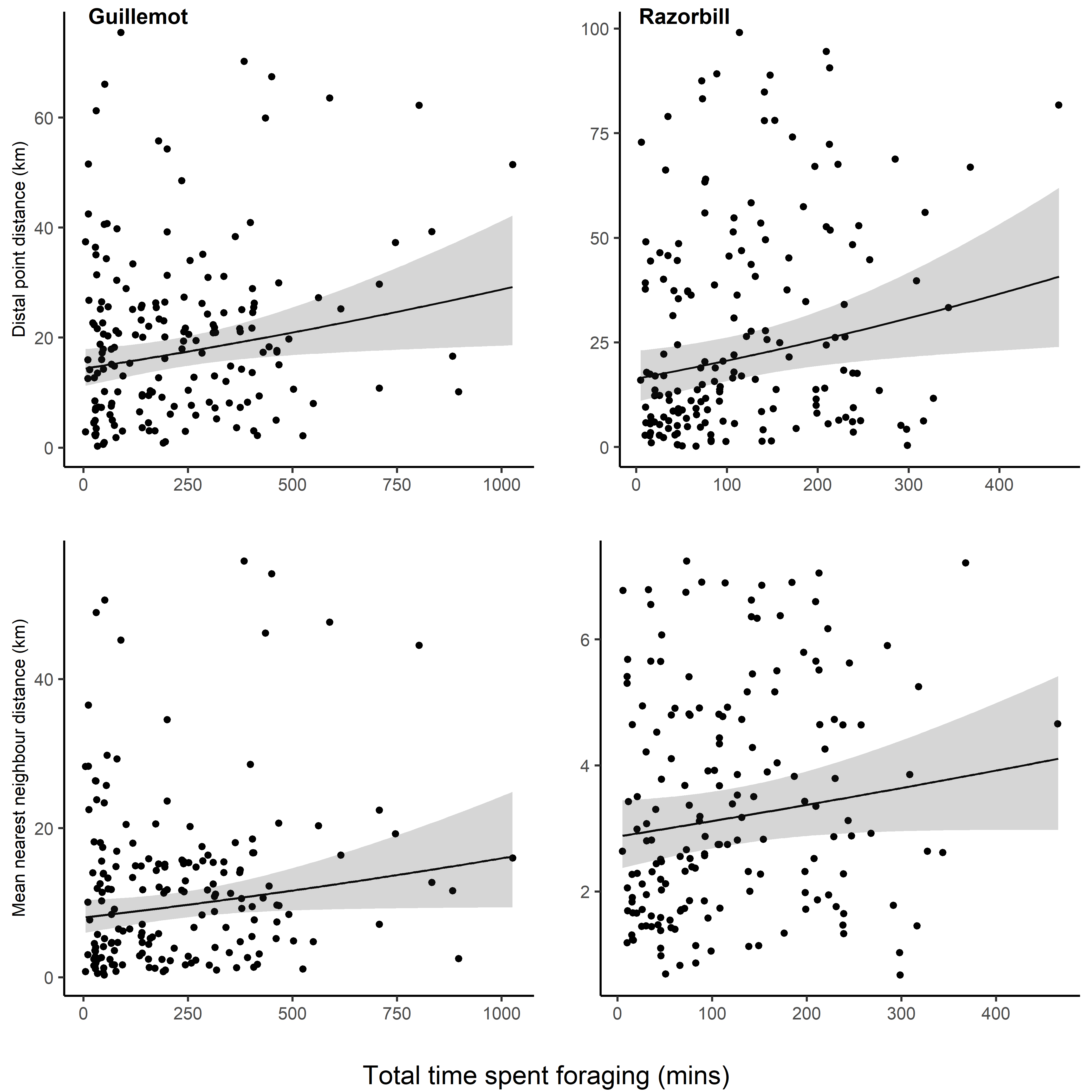


**Figure S5.** Relationship between the total time spent foraging on a trip and the distance between the distal point of that trip and the subsequent trip. Shown are the raw data (points) and model predictions with associated 95% confidence intervals.

**Individual similarity model results**

**Table S7.** Model estimates, standard errors, and P values, from models examining relationships between trip similarity and temporal overlap when examining trips within species only.

|  | **Guillemot** | | | **Kittiwake** | | | **Razorbill** | | | **Puffin** | | | | |
| --- | --- | --- | --- | --- | --- | --- | --- | --- | --- | --- | --- | --- | --- | --- |
|  | Est | SE | P | Est | SE | P | Est | SE | P | Est | SE | | P |  |
| **Nearest neighbour distance** | | | | | | | | | |  | | | | |
| Intercept | 4.002 | 0.114 | <0.001 | 4.325 | 0.219 | <0.001 | 3.789 | 0.240 | <0.001 | 3.519 | | 0.091 | <0.001 | |
| Comparison type (within individual within day) | -0.426 | 0.037 | <0.001 | -0.546 | 0.021 | <0.001 | -0.162 | 0.070 | 0.020 | -0.234 | | 0.155 | 0.132 | |
| Distance between capture sites | 0.048 | 0.019 | 0.011 | -0.040 | 0.010 | <0.001 | -0.015 | 0.037 | 0.678 | 0.028 | | 0.072 | 0.701 | |
|  | Var | SD | P | Var | SD | P | Var | SD | P | Var | | SD | P | |
| Year | 0.082 | 0.287 | <0.001 | 0.287 | 0.535 | <0.001 | 0.379 | 0.616 | <0.001 | 0.000 | | 0.000 | 1 | |
| **Difference between distal points** | | | | | | | | | |  | | | | |
| Intercept | 5.737 | 0.160 | <0.001 | 6.286 | 0.344 | <0.001 | 5.345 | 0.304 | <0.001 | 4.955 | | 0.162 | <0.001 | |
| Comparison type (within individual within day) | -0.773 | 0.052 | <0.001 | -0.693 | 0.031 | <0.001 | -0.299 | 0.095 | 0.002 | -0.637 | | 0.196 | 0.001 | |
| Distance between capture sites | 0.078 | 0.026 | 0.003 | -0.074 | 0.015 | <0.001 | -0.051 | 0.050 | 0.317 | -0.007 | 0.091 | | 0.938 | |
|  | Var | SD | P | Var | SD | P | Var | SD | P | Var | SD | | P | |
| Year | 0.161 | 0.402 | <0.001 | 0.711 | 0.843 | <0.001 | 0.598 | 0.773 | <0.001 | 0.035 | 0.187 | | 0.148 | |
| **Difference in bearing** | | | | | | | | | |  | | | | |
| Intercept | 1.238 | 0.030 | <0.001 | 1.059 | 0.052 | <0.001 | 1.288 | 0.031 | <0.001 | 0.875 | 0.036 | | <0.001 | |
| Comparison type (within individual within day) | -0.055 | 0.016 | <0.001 | -0.269 | 0.007 | <0.001 | -0.083 | 0.028 | 0.003 | 0.038 | 0.061 | | 0.533 | |
| Distance between capture sites | 0.033 | 0.008 | <0.001 | 0.011 | 0.004 | 0.002 | 0.021 | 0.015 | 0.152 | 0.020 | 0.028 | | 0.476 | |
|  | Var | SD | P | Var | SD | P | Var | SD | P | Var | SD | | P | |
| Year | 0.005 | 0.071 | <0.001 | 0.015 | 0.121 | <0.001 | 0.003 | 0.059 | 0.001 | 0.000 | 0.000 | | 1 | |

**Table S8.** Model estimates, standard errors, and P values, from models examining relationships between trip similarity and temporal overlap when examining trips made by different species. Shown are results from models with main effects, only.

|  | **Guillemot** | | | **Kittiwake** | | | **Razorbill** | | | **Puffin** | | | | |
| --- | --- | --- | --- | --- | --- | --- | --- | --- | --- | --- | --- | --- | --- | --- |
|  | Est | SE | P | Est | SE | P | Est | SE | P | Est | SE | | P |  |
| **Nearest neighbour distance** | | | | | | | | | |  | | | | |
| Intercept | 3.636 | 0.125 | <0.001 | 3.933 | 0.222 | <0.001 | 3.649 | 0.269 | <0.001 | 3.472 | | 0.138 | <0.001 | |
| Comparison type (between species within day) | 0.220 | 0.037 | <0.001 | 0.148 | 0.020 | <0.001 | -0.062 | 0.063 | 0.328 | 0.164 | | 0.154 | 0.286 | |
| Species (puffin) | -0.161 | 0.084 | 0.057 | -0.312 | 0.028 | <0.001 | 0.308 | 0.323 | 0.342 | - | | - | - | |
| Species (razorbill) | -0.079 | 0.055 | 0.152 | 0.066 | 0.035 | 0.060 | - | - | - | 0.114 | | 0.460 | 0.803 | |
| Species (kittiwake) | - | - | - | - | - | - | 0.122 | 0.092 | 0.184 | -0.125 | | 0.126 | 0.408 | |
| Distance between capture sites | -0.011 | 0.020 | 0.588 | -0.044 | 0.011 | <0.001 | 0.014 | 0.035 | 0.682 | 0.013 | | 0.084 | 0.875 | |
|  | Var | SD | P | Var | SD | P | Var | SD | P | Var | | SD | P | |
| Year | 0.087 | 0.295 | <0.001 | 0.292 | 0.540 | <0.001 | 0.485 | 0.697 | <0.001 | 0.003 | | 0.059 | 0.819 | |
| **Difference between distal points** | | | | | | | | | |  | | | | |
| Intercept | 5.174 | 0.176 | <0.001 | 5.883 | 0.338 | <0.001 | 5.086 | 0.330 | <0.001 | 4.509 | | 0.163 | <0.001 | |
| Comparison type (between species within day) | 0.456 | 0.050 | <0.001 | 0.199 | 0.030 | <0.001 | 0.020 | 0.084 | 0.807 | 0.497 | | 0.191 | 0.010 | |
| Species (puffin) | -0.607 | 0.113 | <0.001 | -0.643 | 0.040 | <0.001 | 0.059 | 0.429 | 0.891 | - | | - | - | |
| Species (razorbill) | -0.324 | 0.074 | <0.001 | 0.157 | 0.051 | 0.002 | - | - | - | 0.162 | | 0.569 | 0.776 | |
| Species (kittiwake) | - | - | - | - | - | - | 0.027 | 0.122 | 0.028 | 0.016 | | 0.139 | 0.911 | |
| Distance between capture sites | -0.011 | 0.027 | 0.69 | -0.075 | 0.016 | <0.001 | 0.006 | 0.047 | 0.900 | -0.025 | 0.105 | | 0.812 | |
|  | Var | SD | P | Var | SD | P | Var | SD | P | Var | SD | | P | |
| Year | 0.174 | 0.417 | <0.001 | 0.685 | 0.828 | <0.001 | 0.726 | 0.852 | <0.001 | 0.000 | 0.000 | | 1 | |
| **Difference in bearing** | | | | | | | | | |  | | | | |
| Intercept | 1.164 | 0.033 | <0.001 | 0.844 | 0.064 | <0.001 | 1.191 | 0.027 | <0.001 | 0.937 | 0.089 | | <0.001 | |
| Comparison type (between species within day) | 0.102 | 0.016 | <0.001 | 0.372 | 0.007 | <0.001 | -0.066 | 0.026 | 0.009 | 0.120 | 0.061 | | 0.051 | |
| Species (puffin) | 0.066 | 0.036 | 0.064 | -0.007 | 0.010 | 0.499 | 0.019 | 0.130 | 0.882 | - | - | | - | |
| Species (razorbill) | 0.034 | 0.023 | 0.145 | 0.006 | 0.013 | 0.634 | - | - | - | -0.029 | 0.187 | | 0.875 | |
| Species (kittiwake) | - | - | - | - | - | - | 0.007 | 0.036 | 0.851 | 0.001 | 0.067 | | 0.987 | |
| Distance between capture sites | -0.008 | 0.008 | 0.326 | -0.003 | 0.004 | 0.501 | -0.011 | 0.014 | 0.444 | -0.029 | 0.034 | | 0.396 | |
|  | Var | SD | P | Var | SD | P | Var | SD | P | Var | SD | | P | |
| Year | 0.004 | 0.062 | <0.001 | 0.024 | 0.154 | <0.001 | 0.002 | 0.048 | 0.011 | 0.017 | 0.130 | | 0.007 | |

**Table S9**. Model estimates, standard errors, and P values, from models examining relationships between trip similarity and temporal overlap when examining trips made by different species. Shown are results from models including first order interactions between the comparison type (within versus between day) and the species comparison made.

|  | **Guillemot** | | | **Kittiwake** | | | **Razorbill** | | | **Puffin** | | | | |
| --- | --- | --- | --- | --- | --- | --- | --- | --- | --- | --- | --- | --- | --- | --- |
|  | Est | SE | P | Est | SE | P | Est | SE | P | Est | SE | | P |  |
| **Nearest neighbour distance** | | | | | | | | | |  | | | | |
| Intercept | 3.515 | 0.133 | <0.001 | 3.823 | 0.223 | <0.001 | 3.670 | 0.169 | <0.001 | 3.505 | | 0.168 | <0.001 | |
| Comparison type (between species within day) | 0.462 | 0.093 | <0.001 | 0.368 | 0.047 | <0.001 | -0.102 | 0.070 | 0.143 | 0.096 | | 0.246 | 0.697 | |
| Species (puffin) | 0.007 | 0.118 | 0.953 | -0.116 | 0.038 | 0.003 | 0.644 | 0.454 | 0.156 | - | | - | - | |
| Species (razorbill) | 0.063 | 0.075 | 0.399 | -0.069 | 0.050 | 0.166 | - | - | - | -0.212 | | 0.647 | 0.743 | |
| Species (kittiwake) | - | - | - | - | - | - | -0.002 | 0.121 | 0.988 | -0.165 | | 0.169 | 0.357 | |
| Distance between capture sites | -0.012 | 0.020 | 0.551 | -0.043 | 0.011 | <0.001 | 0.011 | 0.036 | 0.766 | 0.009 | | 0.085 | 0.911 | |
| Comparison type * puffin | -0.335 | 0.167 | 0.044 | -0.393 | 0.053 | <0.001 | -0.670 | 0.638 | 0.293 | - | | - | - | |
| Comparison type * razorbill | -0.285 | 0.102 | 0.005 | 0.270 | 0.070 | <0.001 | - | - | - | 0.657 | | 0.915 | 0.473 | |
| Comparison type * kittiwake | - | - | - | - | - | - | 0.248 | 0.156 | 0.112 | 0.082 | | 0.223 | 0.713 | |
|  | Var | SD | P | Var | SD | P | Var | SD | P | Var | | SD | P | |
| Year | 0.087 | 0.295 | <0.001 | 0.293 | 0.541 | <0.001 | 0.483 | 0.695 | <0.001 | 0.004 | | 0.059 | 0.814 | |
| **Difference between distal points** | | | | | | | | | |  | | | | |
| Intercept | 4.804 | 0.184 | <0.001 | 5.700 | 0.340 | <0.001 | 5.112 | 0.330 | <0.001 | 4.554 | | 0.202 | <0.001 | |
| Comparison type (between species within day) | 1.196 | 0.124 | <0.001 | 5.640 | 0.069 | <0.001 | -0.030 | 0.092 | 0.748 | 0.407 | | 0.306 | 0.184 | |
| Species (puffin) | 0.012 | 0.159 | 0.940 | -0.291 | 0.056 | <0.001 | 0.781 | 0.601 | 0.194 | - | | - | - | |
| Species (razorbill) | 0.100 | 0.100 | 0.318 | -0.177 | 0.072 | 0.014 | - | - | - | 0.033 | | 0.804 | 0.967 | |
| Species (kittiwake) | - | - | - | - | - | - | 0.101 | 0.207 | 0.527 | -0.037 | | 0.197 | 0.850 | |
| Distance between capture sites | -0.013 | 0.027 | 0.638 | -0.073 | 0.016 | <0.001 | 0.002 | 0.047 | 0.967 | -0.025 | 0.106 | | 0.810 | |
| Comparison type * puffin | -1.237 | 0.223 | <0.001 | -0.702 | 0.078 | <0.001 | -1.444 | 0.845 | 0.088 | - | - | | - | |
| Comparison type * razorbill | -0.849 | 0.137 | <0.001 | 0.669 | 0.102 | <0.001 | - | - | - | 0.257 | 1.140 | | 0.822 | |
| Comparison type * kittiwake | - | - | - | - | - | - | 0.335 | 0.207 | 0.105 | 0.105 | 0.278 | | 0.704 | |
|  | Var | SD | P | Var | SD | P | Var | SD | P | Var | SD | | P | |
| Year | 0.174 | 0.417 | <0.001 | 0.688 | 0.829 | <0.001 | 0.723 | 0.850 | <0.001 | 0.000 | 0.000 | | 1 | |
| **Difference in bearing** | | | | | | | | | |  | | | | |
| Intercept | 4.804 | 0.184 | <0.001 | 0.885 | 0.065 | <0.001 | 1.183 | 0.028 | <0.001 | 0.881 | 0.097 | | <0.001 | |
| Comparison type (between species within day) | 1.196 | 0.124 | <0.001 | 0.289 | 0.017 | <0.001 | -0.051 | 0.028 | 0.073 | 0.233 | 0.098 | | 0.018 | |
| Species (puffin) | 0.012 | 0.159 | 0.940 | -0.063 | 0.014 | <0.001 | 0.050 | 0.183 | 0.785 | - | - | | - | |
| Species (razorbill) | 0.100 | 0.100 | 0.318 | -0.020 | 0.018 | 0.270 | - | - | - | -0.021 | 0.260 | | 0.936 | |
| Species (kittiwake) | - | - | - | - | - | - | 0.047 | 0.048 | 0.320 | 0.067 | 0.081 | | 0.409 | |
| Distance between capture sites | -0.013 | 0.027 | 0.638 | -0.003 | 0.004 | 0.507 | -0.009 | 0.014 | 0.527 | -0.031 | 0.034 | | 0.369 | |
| Comparison type * puffin | -1.237 | 0.223 | <0.001 | 0.113 | 0.019 | <0.001 | -0.063 | 0.258 | 0.809 | - | - | | - | |
| Comparison type * razorbill | -0.849 | 0.137 | <0.001 | 0.051 | 0.025 | 0.042 | - | - | - | -0.016 | 0.363 | | 0.966 | |
| Comparison type * kittiwake | - | - | - | - | - | - | -0.081 | 0.063 | 0.200 | -0.131 | 0.089 | | 0.141 | |
|  | Var | SD | P | Var | SD | P | Var | SD | P | Var | SD | | P | |
| Year | 0.004 | 0.062 | <0.001 | 0.024 | 0.154 | <0.001 | 0.002 | 0.048 | 0.010 | 0.017 | 0.130 | | 0.007 | |

**Table S10.** Model estimates, standard errors, and P values, from models examining relationships between trip similarity and whether individuals overlapped at the colony or not prior to trip departure.

|  | **Guillemot** | | | **Kittiwake** | | | **Razorbill** | | | **Puffin** | | | | |
| --- | --- | --- | --- | --- | --- | --- | --- | --- | --- | --- | --- | --- | --- | --- |
|  | Est | SE | P | Est | SE | P | Est | SE | P | Est | SE | | P |  |
| **Nearest neighbour distance** | | | | | | | | | |  | | | | |
| Intercept | 3.500 | 0.173 | <0.001 | 3.731 | 0.237 | <0.001 | 3.741 | 0.292 | <0.001 | 3.288 | | 0.134 | <0.001 | |
| Overlap at colony (true) | 0.035 | 0.103 | 0.736 | -0.079 | 0.086 | 0.359 | -0.042 | 0.210 | 0.84 | -0.463 | | 0.156 | 0.003 | |
|  | Var | SD | P | Var | SD | P | Var | SD | P | Var | | SD | P | |
| Year | 0.074 | 0.272 | 0.365 | 0.202 | 0.449 | 0.127 | 0.316 | 0.562 | 0.010 | 0.000 | | 0.000 | 1 | |
| Day:Year | 0.426 | 0.653 | <0.001 | 0.434 | 0.659 | <0.001 | 0.122 | 0.349 | 0.100 | 0.259 | | 0.509 | <0.001 | |
| **Difference between distal points** | | | | | | | | | |  | | | | |
| Intercept | 4.695 | 0.269 | <0.001 | 5.460 | 0.317 | 0.003 | 5.184 | 0.397 | <0.001 | 4.418 | | 0.147 | <0.001 | |
| Overlap at colony (true) | 0.098 | 0.132 | 0.457 | -0.042 | 0.123 | 0.732 | -0.118 | 0.286 | 0.68 | -0.510 | | 0.186 | 0.006 | |
|  | Var | SD | P | Var | SD | P | Var | SD | P | Var | | SD | P | |
| Year | 0.239 | 0.489 | 0.176 | 0.346 | 0.588 | 0.318 | 0.605 | 0.778 | 0.488 | 0.000 | | 0.000 | 1 | |
| Day:Year | 0.936 | 0.968 | <0.001 | 0.761 | 0.872 | <0.001 | 0.092 | 0.304 | 0.004 | 0.272 | | 0.522 | <0.001 | |
| **Difference in bearing** | | | | | | | | | |  | | | | |
| Intercept | 1.188 | 0.099 | <0.001 | 0.857 | 0.062 | 0.002 | 1.132 | 0.077 | <0.001 | 0.854 | 0.047 | | <0.001 | |
| Overlap at colony (true) | 0.014 | 0.044 | 0.749 | -0.026 | 0.023 | 0.256 | -0.054 | 0.074 | 0.471 | -0.119 | 0.056 | | 0.037 | |
|  | Var | SD | P | Var | SD | P | Var | SD | P | Var | SD | | P | |
| Year | 0.047 | 0.216 | 0.007 | 0.016 | 0.127 | 0.231 | 0.004 | 0.064 | 0.867 | 0.000 | 0.000 | | 1 | |
| Day:Year | 0.053 | 0.231 | <0.001 | 0.011 | 0.107 | <0.001 | 0.052 | 0.227 | 0.003 | 0.030 | 0.173 | | <0.001 | |


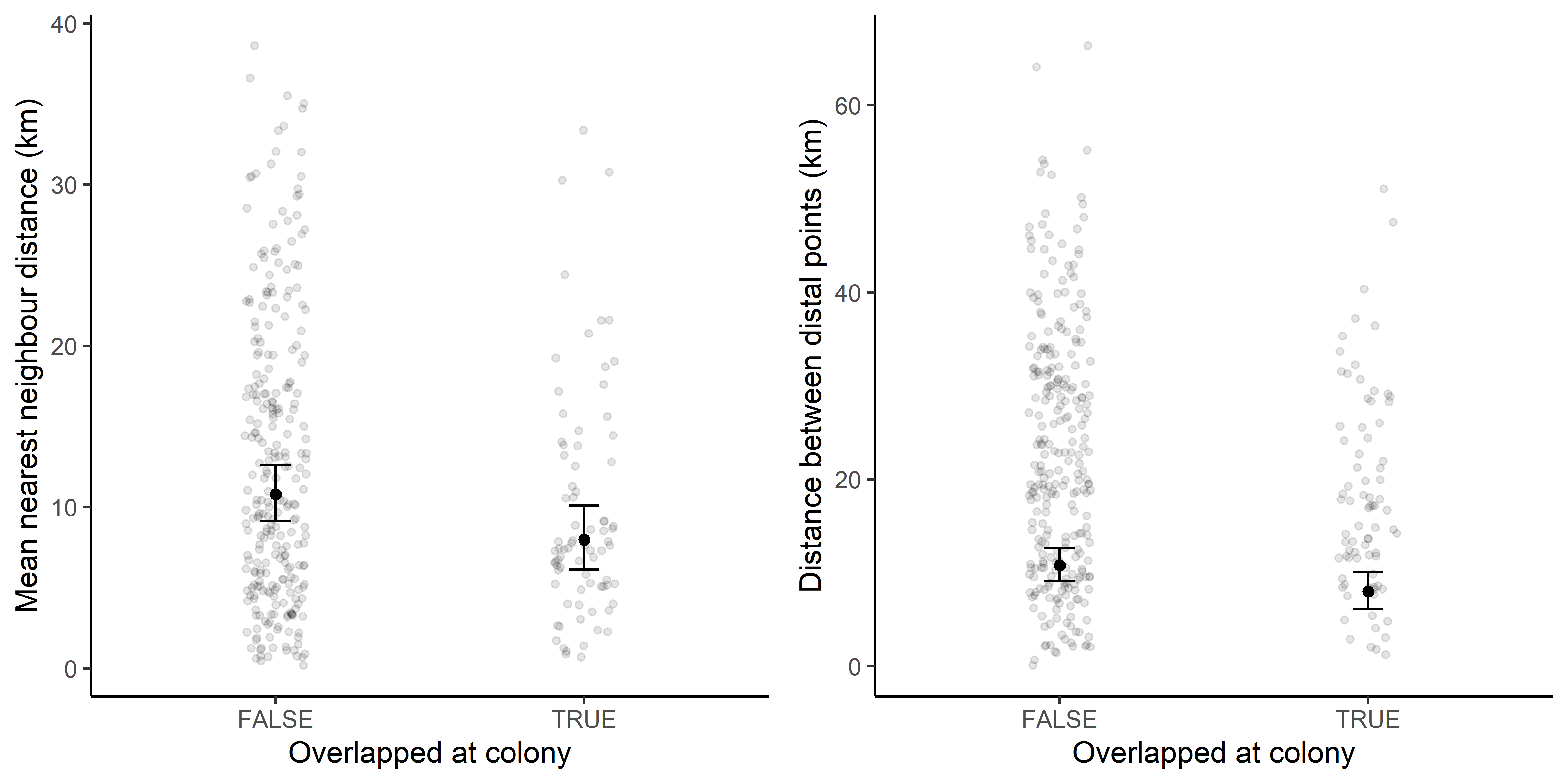


**Figure S6.** Relationship between the mean nearest neighbour distance between a pair of trips and whether the individuals overlapped at the colony prior to departure for Puffins. Shown are the raw data and model predictions with associated 95% confidence intervals.

**Table S11.** Model estimates, standard errors, and P values, from models examining relationships between trip similarity and the degree to which individuals overlapped out at sea when making their foraging trips.

|  | **Guillemot** | | | **Kittiwake** | | | **Razorbill** | | | **Puffin** | | | | |
| --- | --- | --- | --- | --- | --- | --- | --- | --- | --- | --- | --- | --- | --- | --- |
|  | Est | SE | P | Est | SE | P | Est | SE | P | Est | SE | | P |  |
| **Nearest neighbour distance** | | | | | | | | | |  | | | | |
| Intercept | 3.664 | 0.175 | <0.001 | 3.854 | 0.156 | 0.014 | 3.264 | 0.308 | <0.001 | 3.338 | | 0.124 | <0.001 | |
| Amount of overlap | -0.200 | 0.032 | <0.001 | -0.218 | 0.022 | <0.001 | -0.251 | 0.075 | <0.001 | -0.378 | | 0.067 | <0.001 | |
| Distance between capture sites | 0.111 | 0.033 | 0.001 | -0.021 | 0.024 | 0.368 | 0.072 | 0.084 | 0.394 |  | |  |  | |
|  | Var | SD | P | Var | SD | P | Var | SD | P | Var | | SD | P | |
| Year | 0.151 | 0.388 | 0.017 | 0.068 | 0.260 | 0.334 | 0.538 | 0.734 | <0.001 | 0.000 | | 0.000 | 1 | |
| Identity:Year | 0.287 | 0.536 | <0.001 | 0.287 | 0.536 | <0.001 | 0.182 | 0.427 | 0.002 | 0.180 | | 0.425 | <0.001 | |
| **Difference between distal points** | | | | | | | | | |  | | | | |
| Intercept | 5.106 | 0.216 | <0.001 | 5.592 | 0.172 | <0.001 | 4.611 | 0.407 | <0.001 | 4.468 | | 0.153 | <0.001 | |
| Amount of overlap | -0.050 | 0.045 | 0.27 | -0.240 | 0.033 | <0.001 | -0.253 | 0.112 | 0.024 | -0.281 | | 0.084 | <0.001 | |
| Distance between capture sites | 0.205 | 0.047 | <0.001 | -0.068 | 0.035 | 0.055 | 0.161 | 0.125 | 0.199 |  | |  |  | |
|  | Var | SD | P | Var | SD | P | Var | SD | P | Var | | SD | P | |
| Year | 0.216 | 0.465 | 0.036 | 0.054 | 0.233 | 0.466 | 0.904 | 0.951 | 0.002 | 2.900× 10^-10^ | | 1.703× 10^-5^ | 1 | |
| Identity:Year | 0.484 | 0.696 | <0.001 | 0.504 | 0.710 | <0.001 | 0.351 | 0.592 | 0.007 | 0.273 | | 0.523 | <0.001 | |
| **Difference in bearing** | | | | | | | | | |  | | | | |
| Intercept | 1.147 | 0.044 | <0.001 | 0.729 | 0.024 | <0.001 | 1.035 | 0.045 | <0.001 | 0.712 | 0.101 | | 0.010 | |
| Amount of overlap | -0.045 | 0.015 | 0.003 | -0.032 | 0.006 | <0.001 | -0.116 | 0.031 | <0.001 | -0.082 | 0.030 | | 0.006 | |
| Distance between capture sites | 0.078 | 0.015 | <0.001 | -0.002 | 0.007 | 0.793 | 0.061 | 0.032 | 0.062 |  |  | |  | |
|  | Var | SD | P | Var | SD | P | Var | SD | P | Var | SD | | P | |
| Year | 0.007 | 0.081 | 0.262 | 0.001 | 0.034 | 0.296 | 0.003 | 0.055 | 0.669 | 0.022 | 0.149 | | 0.095 | |
| Identity:Year | 0.026 | 0.161 | <0.001 | 0.006 | 0.077 | <0.001 | 0.015 | 0.121 | 0.129 | 0.022 | 0.149 | | <0.001 | |
